# Anti-integrin α_v_ therapy improves cardiac fibrosis after myocardial infarction by blunting cardiac PW1^+^ stromal cells

**DOI:** 10.1038/s41598-020-68223-8

**Published:** 2020-07-09

**Authors:** Marion Bouvet, Olivier Claude, Maguelonne Roux, Dan Skelly, Nihar Masurkar, Nathalie Mougenot, Sophie Nadaud, Catherine Blanc, Clément Delacroix, Solenne Chardonnet, Cédric Pionneau, Claire Perret, Elisa Yaniz-Galende, Nadia Rosenthal, David-Alexandre Trégouët, Giovanna Marazzi, Jean-Sébastien Silvestre, David Sassoon, Jean-Sébastien Hulot

**Affiliations:** 1 Université de Paris, PARCC, INSERM, 56 Rue Leblanc, 75015 Paris, France; 2 Sorbonne Université, UPMC Univ Paris 06, INSERM, Institute of Cardio Metabolism and Nutrition (ICAN), Paris, France; 3 The Jackson Laboratory, Bar Harbor, ME USA; 4 Sorbonne Université, UPMC Univ Paris 06, PECMV, UMS28, Paris, France; 5 Sorbonne Université, Inserm, UMS Omique, Plateforme Post-génomique de la Pitié-Salpêtrière, P3S, 75013 Paris, France; 6 INSERM UMR_S 1219, Bordeaux Population Health Research Center, University of Bordeaux, Bordeaux, France

**Keywords:** Cardiovascular biology, Heart failure, Adult stem cells

## Abstract

There is currently no therapy to limit the development of cardiac fibrosis and consequent heart failure. We have recently shown that cardiac fibrosis post-myocardial infarction (MI) can be regulated by resident cardiac cells with a fibrogenic signature and identified by the expression of PW1 (*Peg3*). Here we identify αV-integrin (CD51) as an essential regulator of cardiac PW1^+^ cells fibrogenic behavior. We used transcriptomic and proteomic approaches to identify specific cell-surface markers for cardiac PW1^+^ cells and found that αV-integrin (CD51) was expressed in almost all cardiac PW1^+^ cells (93% ± 1%), predominantly as the αVβ1 complex. αV-integrin is a subunit member of the integrin family of cell adhesion receptors and was found to activate complex of latent transforming growth factor beta (TGFβ at the surface of cardiac PW1^+^ cells. Pharmacological inhibition of αV-integrin reduced the profibrotic action of cardiac PW1^+^CD51^+^ cells and was associated with improved cardiac function and animal survival following MI coupled with a reduced infarct size and fibrotic lesion. These data identify a targetable pathway that regulates cardiac fibrosis in response to an ischemic injury and demonstrate that pharmacological inhibition of αV-integrin could reduce pathological outcomes following cardiac ischemia.

## Introduction

Heart failure (HF) remains a leading cause of mortality and hospitalization worldwide and represents a heavy financial burden on health care systems^1–6^. Current pharmacological treatments limit the peripheral consequences of cardiac dysfunction, but therapeutic approaches that impact primary adverse cardiac remodeling at the myocardial level are limited. While the etiology of HF is diverse, HF is typically associated with a range of physiological and morphological changes including fibrosis within the myocardium^7–9^. Cardiac fibrosis is characterized with the excessive production and deposition of extracellular matrix (ECM) proteins within the myocardium that results in normal tissue architecture disruption, reduced tissue compliance, and mechanical and electrical dysfunction, consequently leading to HF^10,11^. The mechanisms underlying cardiac fibrosis are incompletely understood, thereby impeding the development of effective anti-fibrotic therapeutics to complement the current therapies for HF^9,11,12^.

Activated cardiac fibroblasts are essential for the production of ECM proteins that accumulate during cardiac fibrosis; however, recent studies have established that cardiac fibroblasts represent a heterogeneous cell population^10–14^. The exact nature of activated fibroblasts and consequently the sources of cardiac fibrosis remain unclear^9,12^*.* Different mechanisms underlying fibrosis have been reported including the activation and proliferation of resident fibroblasts^15^, transformation of endothelial and/or epicardial cells after injury through endothelial-mesenchymal transition and epithelial–mesenchymal transition respectively^16,17^, and migration of hematopoietic bone marrow-derived cells and perivascular cells^18^. Another model proposes the activation of tissue-resident progenitor populations in response to stress that serves as a major cellular source of organ fibrosis, including the heart. In a recent study, we described a novel population of cardiac stromal cells that resides in the myocardium and exhibits a fibrogenic fate in response to cardiac ischemic injury^19^. This population was identified based on the expression of the pan-stem cell marker, Pw1/Peg3 (referred hereafter as PW1)^20,21^, using a transgenic Pw1-beta galactosidase (β-gal) reporter mouse model (Pw1^nLacZ^). We found that at least ~ 22% of fibroblasts in the fibrotic region of ischemic hearts were derived from PW1-expressing cells, demonstrating that cardiac PW1^+^ cells directly contribute to cardiac fibrosis. However, the exact pathways mediating the fibrogenic activity of cardiac PW1^+^ cells remain to be elucidated.

PW1 is a zinc finger transcription factor and cell stress mediator, expressed in the nucleus and cytosol of cells. Therefore, we set out to identify specific cell surface markers for cardiac PW1^+^ cells under physiological and pathological situations using a combination of transcriptomics and proteomics approaches. This combined approach led to the identification of αV-integrin (CD51, encoded by *Itgav*) which is detectable in > 90% of cardiac PW1^+^ cells. αV-integrin is a subunit member of the integrin family of cell adhesion receptors and previous studies show that these molecules are central mediators of organ fibrosis through the TGF-beta signaling pathway^22,23^. We found that αV-integrin is directly involved in directing the fibrogenic cell fate of cardiac PW1^+^ cells and specifically the blockade of αV-integrin results in a marked reduction in cardiac PW1^+^ fibrotic activation as well as in cardiac fibrosis post-myocardial infarction (MI) in vivo.

## Results

### Characterization of the cardiac PW1^+^ cell membranome

**Figure 1:**
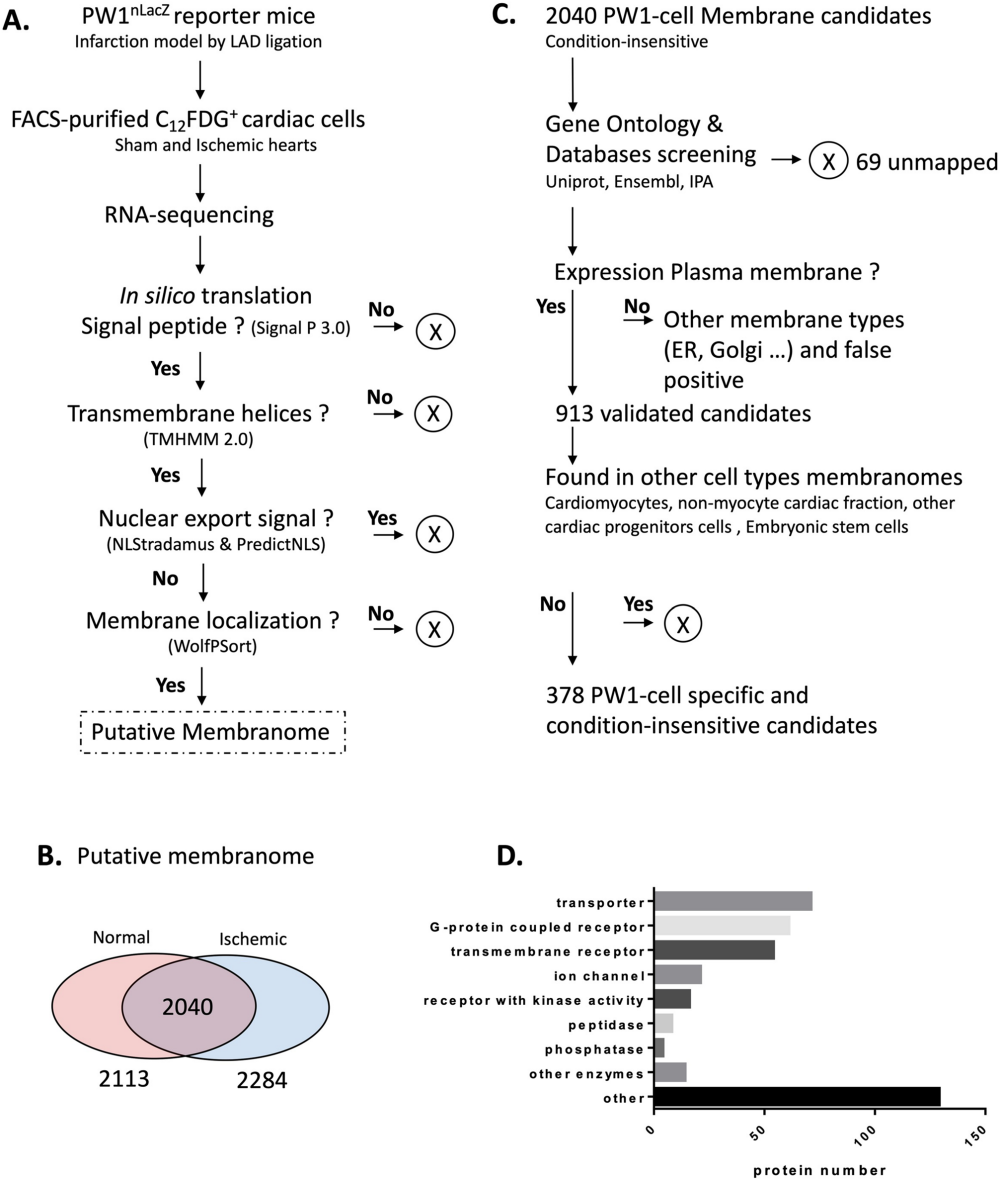
Bioinformatic membranome analysis. (**A**) Experimental strategy and bioinformatic algorithm to identify putative membrane proteins in FACS-purified PW1^+^ cardiac cells. (**B**) PW1^+^ cell putative membranome: 2,113 and 2,284 sequences were identified in cells from sham and ischemic hearts, respectively. A total of 2,040 sequences were identified under both conditions. (**C**) Experimental strategy to validate the newly identified candidates specifically expressed in the plasma membrane of PW1^+^ cardiac cells. (**D**) Molecular function of the 378 PW1^+^ cell-specific membrane proteins. ER, endoplasmic reticulum, FACS, fluorescence-activated cell sorting.

We first characterized the transcriptome profile of cardiac PW1^+^ cells by RNA-seq. We used PW1^nLacZ^ reporter mice to fluorescence-activated cell sorting (FACS)-isolate cardiac PW1^+^ cells from sham-operated hearts and post-MI hearts obtained 7 days after left anterior descending artery (LAD) ligation, as previously described^19,20^. We then filtered, aligned, quality-controlled RNA-seq output files to predict amino acid sequences of the corresponding genes, and applied a series of bioinformatic algorithms to identify putative membrane proteins (Fig. 1A). We considered proteins with a predicted N-terminal endoplasmic reticulum targeting signal peptide and predicted transmembrane domains but without intracellular localization signals (i.e., no endoplasmic reticulum retention signal motif and no mitochondrial targeting peptide or nuclear export signal). By progressive filtering, we generated a list of 2,040 candidates expressed in cardiac PW1^+^ cell membranes under both normal and ischemic conditions (Fig. 1B). The characteristics of these 2,040 candidates were subsequently screened using available databases to identify 913 candidates expressed in the plasma membrane of cardiac PW1^+^ cells (Fig. 1C). In parallel, we used the same strategy to define the surface membranome of other cell types, including cardiomyocytes (CMs), non-myocytes heart fractions, and mouse embryonic stem cells, and performed a final screen to limit our list to 378 candidates, which were present in the datasets obtained from cardiac PW1^+^ cells (Fig. 1C). Functional enrichment analyses of these cardiac PW1^+^-specific and condition-insensitive cell surface markers allowed us to identify an important number of transmembrane receptors and transporters, and a smaller proportion of ion channels and enzymes (Fig. 1D), although the molecular function for the majority of the identified candidates was undefined.

**Figure 2:**
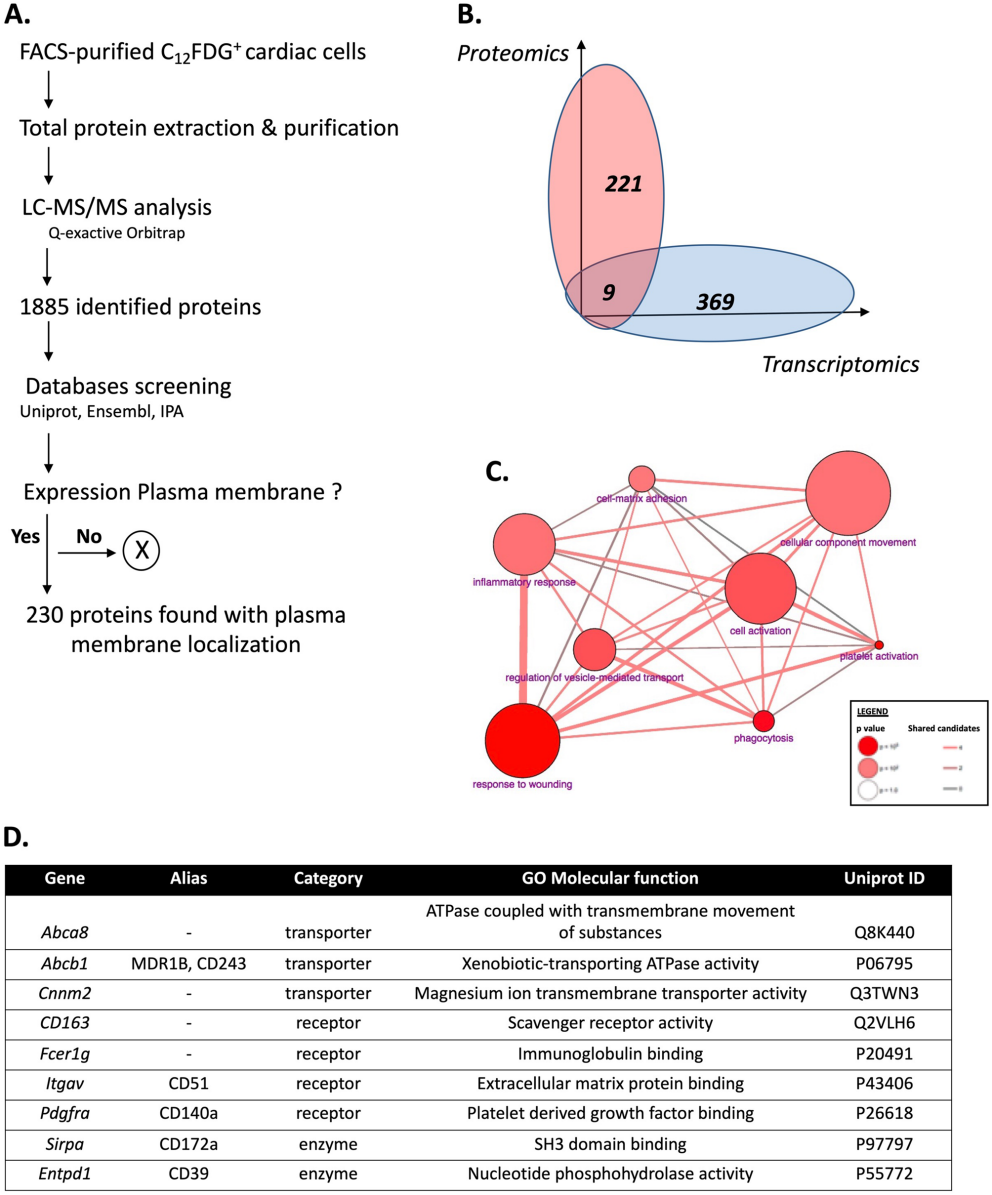
Proteomic cross-validation of cardiac PW1^+^ new cell surface markers. (**A**) Experimental strategy to identify the membrane proteins observed in the full proteome of FACS-purified PW1^+^ cardiac cells. (**B**) Comparison between the two strategies cross-validates the expression of nine candidates. (**C**) Overlap between gene ontology categories of the newly identified cell surface markers for cardiac PW1^+^ cells obtained with ConsensusDB. (**D**) List of genes, IDs, aliases, categories and molecular functions of the nine candidates.

In parallel to the transcriptomic approach, we performed a proteomic analysis of FACS-isolated cardiac PW1^+^ cells using mass spectrometry (MS) (Fig. 2A). The expression of 1,885 membrane proteins was found and their characteristics were subsequently screened using available databases to identify 230 proteins with confirmed plasma membrane location. We then made a direct comparison between the transcriptomic and proteomic datasets and cross-identified nine candidates obtained with both approaches (Fig. 2B). These nine cell surface proteins comprised three transporters, four receptors, and two enzymes and were primarily involved in cell motility, adhesion to the matrix, inflammatory response and response to wounding (Fig. 2C,D). Five of these candidates have been previously described in non-cardiac cells as plasma membrane clusters of differentiation (i.e., CD51-*Itgav*, CD140a-*Pdgfra*, CD172a-*Sirpa*, CD39-*Entpd1,* and CD163) and were thus chosen for further analysis.

### αV-integrin (CD51) is highly expressed on cardiac PW1^+^ cells

**Figure 3:**
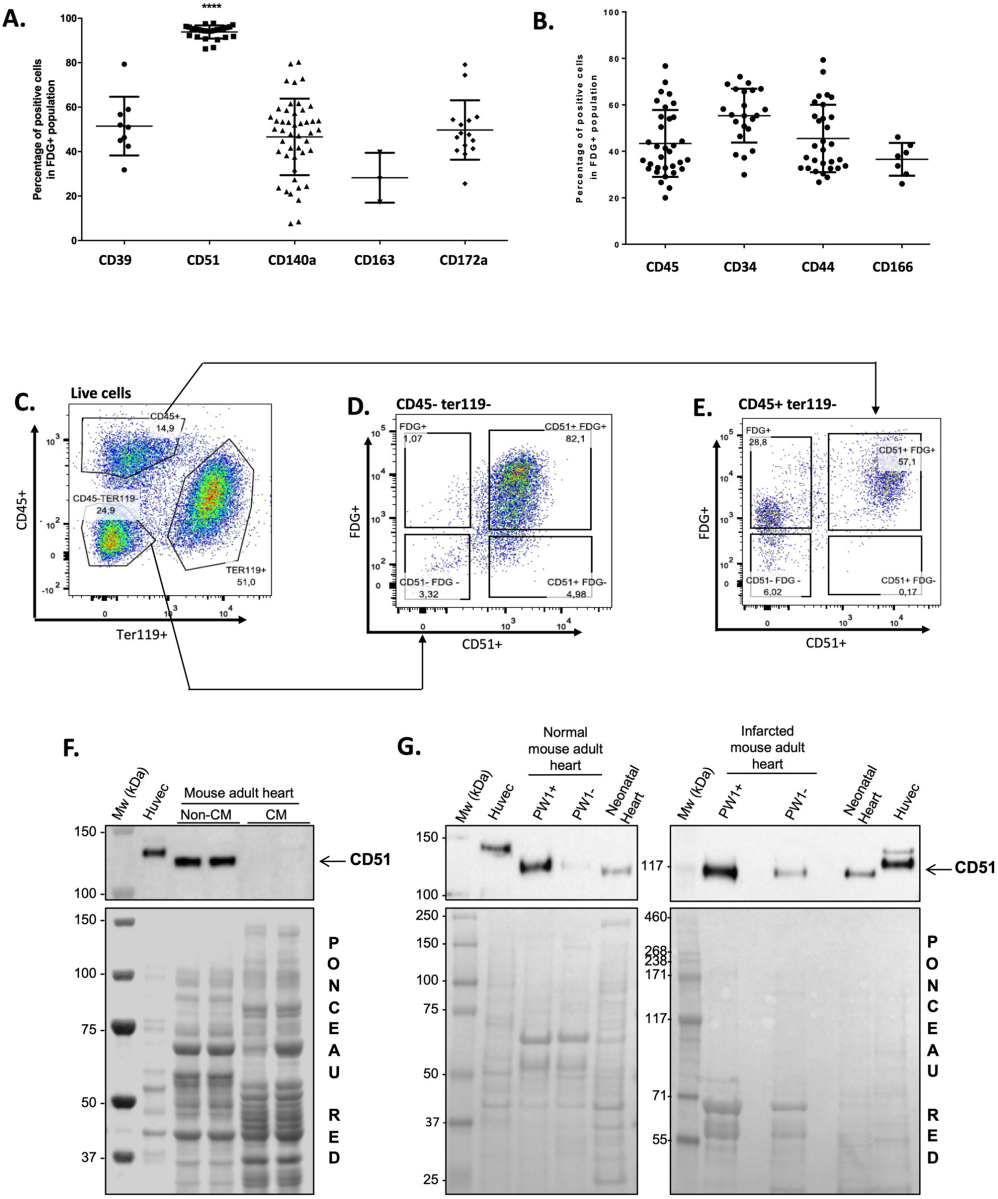
Itgav (CD51) is identified as a sensitive cell surface marker of cardiac PW1^+^ cells. (**A**,**B**) Analysis of the expression of the newly identified cell surface markers (**A**) or typical cell surface markers (**B**) in PW1^+^ cardiac cells by flow cytometry. N > 10. Graphs show mean ± SD. (**C**) Analysis strategy to isolate Ter119^−^ cells. (**D**,**E**) CD51 and FDG expression in CD45^−^Ter119^−^ cells (**D**) and CD45^+^Ter119^−^ cells (**E**). (**F**) Cardiomyocytes (CM) and non-CM cells were isolated from wild-type adult mouse hearts and analyzed by western blotting for the expression of CD51. Proteins from HUVEC were used as positive controls. Ponceau red staining showed equal protein loading. (**G**) PW1^+^ and PW1^−^ fractions were FACS-isolated from sham (left panel) and ischemic (7 days post-MI, right panel) hearts of adult PW1^nLacZ^ mice and pooled to analyze the expression of CD51 by western blotting. Proteins from HUVEC and total neonatal hearts were used as controls. *kDa* kilodalton, *Mw* molecular weight.

The expression of these five cell surface markers was analyzed by cytometry in 5-dodecanoylaminofluorescein di-β-d-galactopyranoside positive (C_12_FDG^+)^ cells isolated from PW1^nLacZ^ reporter mouse hearts. We observed that 92.98% ± 1.01% of cardiac PW1^+^ cells express αV-integrin (CD51) (Fig. 3A); the remaining four markers, as well as the typical adult stem cell markers (i.e., CD44, CD34, and CD166), were expressed in a lower proportion of cardiac PW1^+^ cells (i.e., around 50%; Fig. 3B). In order to eliminate hematopoietic circulating cells from the resident stromal cells, further analyses of sorted CD45^−^Ter119^-^ cardiac cells revealed the strong co-expression of the PW1 reporter and αV-integrin (CD51) expression, confirming that the resident cardiac PW1^+^ cells exhibit high level expression of αV-integrin (Fig. 3C,D). The FDG^+^CD45^−^Ter119^−^ cardiac cells were also characterized with high level expression of CD140a (i.e., platelet-derived growth factor receptor alpha [PDGFRα]), another candidate from our membranome study (Fig. 2D; SI Fig. S1A). Reciprocally, αV-integrin (CD51) expression was observed in the majority of FDG^+^CD45^+^Ter119^−^ cardiac cells (Fig. 3E), which were negative for PDGFRα expression (SI Fig. S1B). We then analyzed αV-integrin (CD51) protein expression in CMs and non-myocytes fractions freshly isolated from normal mouse hearts. HUVEC cells were used as positive controls^24^. As a result, CD51 expression was exclusively detected in the non-CM fraction (Fig. 3F; SI Fig. S2A). We FACS-sorted PW1^+^ and PW1^−^ cell fractions from normal and ischemic mouse hearts and detected CD51 expression mainly in the cardiac PW1^+^ cells (Fig. 3G; SI Fig. S2B), consistent with the results of transcriptomic, proteomic, and cytometry analyses. Western blot analysis further confirmed the significant increase in αV-integrin (CD51) expression in ischemic hearts, more specifically in the infarct zone (SI Fig. S3). This observation is in line with the significant increase in cardiac PW1^+^ cell population post-MI, predominantly in the infarct area^19^. These results indicate that αV-integrin (CD51) is expressed in almost all cardiac PW1^+^ cells, and predominantly found in the cells expressing PW1 in the myocardium.

### Cardiac PW1^+^ and CD51^+^ cells express fibrogenic genes

**Figure 4:**
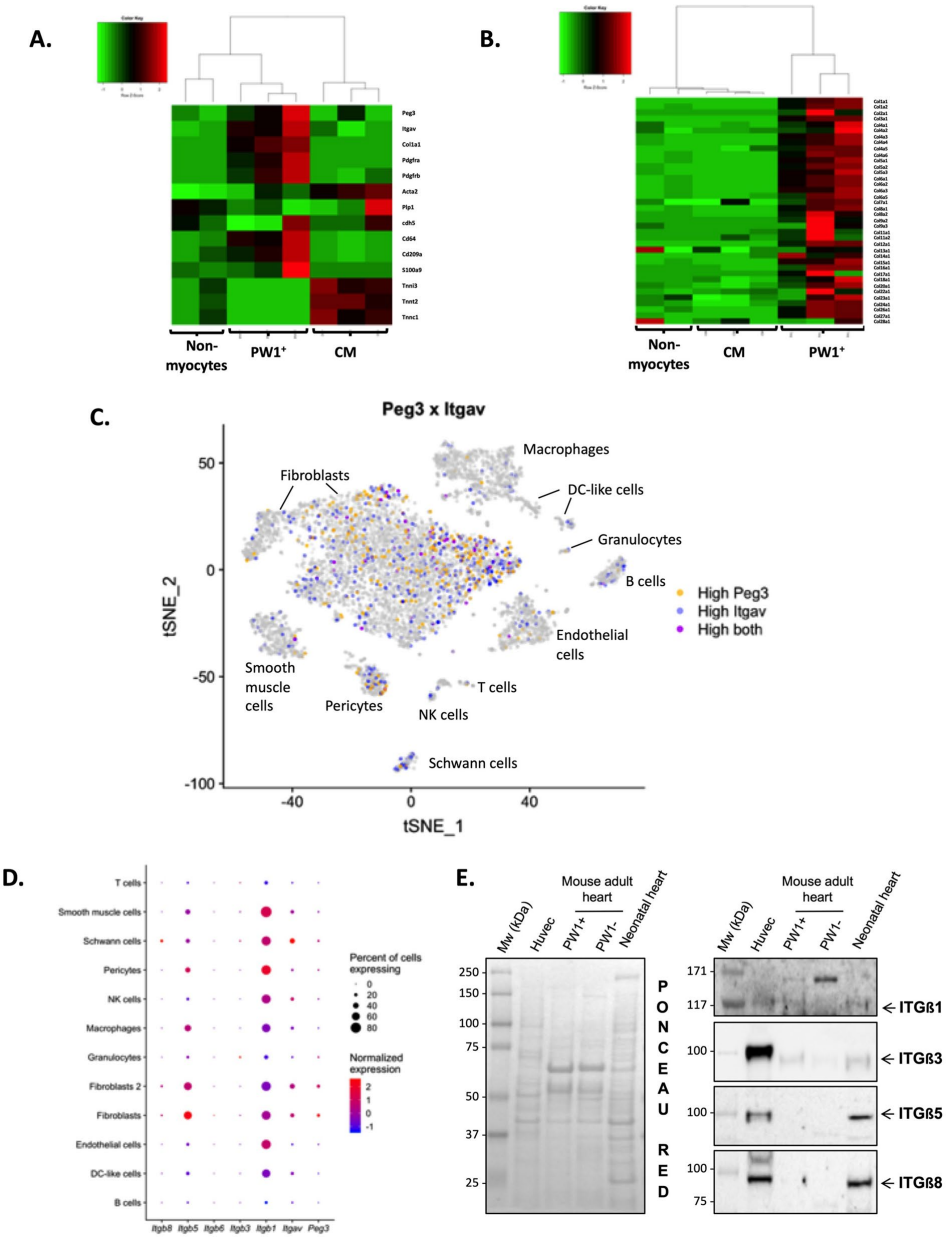
Cardiac PW1^+^CD51^+^ cells express a fibrogenic program. (**A**) Hierarchical clustering based on the expression of Peg3, Itgav, and known cell type markers in FACS-isolated PW1^+^ cells, cardiomyocytes, and non-myocyte fractions from normal mouse hearts. (**B**) Hierarchical clustering showing high expression of collagen genes in PW1^+^ cells. (**C**) Expression of Peg3 (yellow) and Itgav (blue) in major cardiac cell populations identified after unsupervised clustering of single-cell RNA-sequencing data as previously reported^25^. Each point depicts a single cell. Principal component analysis and t-distributed stochastic neighbor embedding (t-SNE) were performed to provide a 2D mapping of clusters. Joint expression of Peg3 and Itgav within single cells is showed in purple. (**D**) The abundance of *Peg3* and *Itgav* mRNAs and those encoding Itgβ1, Itgβ3, Itgβ5, and Itgβ8 according to cell types identified using single-cell RNA-sequencing from normal mouse hearts. (**E**) Western blots for ITGβ1, ITGβ3, ITGβ5, and ITGβ8 in PW1^+^ versus PW1^−^ cardiac fractions. Proteins from HUVEC cells and neonatal mouse heart were used as positive controls. Ponceau Red staining showed equal protein loading.

To better characterize the PW1^+^CD51^+^ cardiac cell population, we first investigated the RNA-seq data for the expression of known markers of major cell types^25^, including fibroblasts (*col1a1, Pdgfra*), pericytes (*Pdgfrb*), smooth muscle cells (*Acta2*), Schwann cells (*plp1*), myeloid leukocytes (*cd64/Fcgr1, cd209a*, *S100a9*) and CMs (*tnni3, tnnt2, tnnc1*) as well as *Peg3* (encoding PW1) and *Itgav* (encoding CD51). In comparison to CMs and non-CMs, FACS-isolated cardiac PW1^+^ cells from normal mouse hearts showed higher expression of *Itgav* and a hybrid molecular signature of fibroblasts, macrophages and to a slightly lower extent of pericytes (Fig. 4A). Hierarchical clustering further revealed the higher expression level of collagen genes in cardiac PW1^+^ cells than in other cell populations (Fig. 4B), suggesting that cardiac PW1^+^CD51^+^ cells exhibit fibroblast-like transcriptional signature.

To further confirm the cellular identity of cardiac PW1^+^ CD51^+^ cells, we analyzed *Peg3* and *Itgav* expressions in single-cell RNA-seq profiles of murine cardiac non-myocytes cells^25^. As a result, we observed that both markers did not specifically tag any cell population but were primarily expressed in fibroblasts, pericytes and Schwann cells at high levels (Fig. 4C); low level expression was detected in other cell populations such as smooth muscle cells, and endothelial cells. Per-cell gene expression measurements are sparse in droplet single-cell RNA-seq data, making it difficult to infer gene co-expression within single cells with high confidence. Nevertheless, joint expression of both markers was the highest in fibroblasts (Fig. 4C), supporting our previous report (Fig. 4A,B and^19^) that cardiac PW1^+^CD51^+^ cells are predominantly identified as fibroblasts or cells involved in fibrotic tissue remodeling.

### Cardiac PW1^+^ cells express different αV-containing integrins

αV-integrin (CD51) belongs to the family of integrins that are transmembrane receptors which act as bridges for cell–ECM connections and cell–cell interactions. αV-integrin subunits can combine to beta subunits to form different integrin combinations depending on specific cell types. To investigate whether there exists an integrin complex specific to PW1^+^ cardiac stromal cells, we assessed the expression of *Peg3*, *Itgav,* and genes encoding beta subunits (including *Itgb1, Itgb3, Itgb5*, and *Itgb8*) in different murine non-myocytes cardiac cells using single-cell RNA seq data (Fig. 4D). We found that the mRNA expression level of *Itgb1* was higher among all other beta subunits and in almost all cell types including fibroblasts, which showed the highest expression levels of *Peg3* and *Itgav*. Thus, αVβ1 complex is predominantly associated with cardiac PW1^+^ cells. Western blotting for cardiac cell fractions (SI Fig. S4A) confirmed the presence of ITGβ1, ITGβ3, ITGβ5, and ITGβ8 in non-CMs. The expression of ITGβ1 and ITGβ3, but not ITGβ5 and ITGβ8, on cardiac PW1^+^ cells was further confirmed with western blotting (Fig. 4E; SI Fig. S4B). In addition, ITGβ1 and ITGβ3 were overexpressed in the infarct areas of post-MI hearts as compared to controls (SI Figs. S5, S6). Overall, these results confirm the high expression of αV-integrin (CD51) and ITGβ1 and ITGβ3 on cardiac PW1^+^ cells, and suggest the presence of complexes consisting of αVβ1 and αVβ3 heterodimers.

### αV-integrin blockade reduces the profibrotic ability of cardiac PW1^+^ cells

Recent studies have shown that αV-integrin functions as the central mediator of organ fibrosis through TGFβ activation^22,23^, in particular αVβ1 integrin. As our data indicate the presence of the αVβ1 complex on cardiac PW1^+^ cells, we investigated whether αV-integrin (CD51) blockade could directly affect the contribution of cardiac PW1^+^ cells to fibrosis. αV-integrin recognizes and binds the tripeptide sequence RGD (arginine, glycine, aspartic acid), a key motif for cell–cell and cell–ECM interactions. Based on this motif, a cyclic RGD pentapeptide was developed (called cilengitide) and shown to inhibit αV-integrin activity, including αVβ1, αVβ3, and αVβ5 activity.

**Figure 5:**
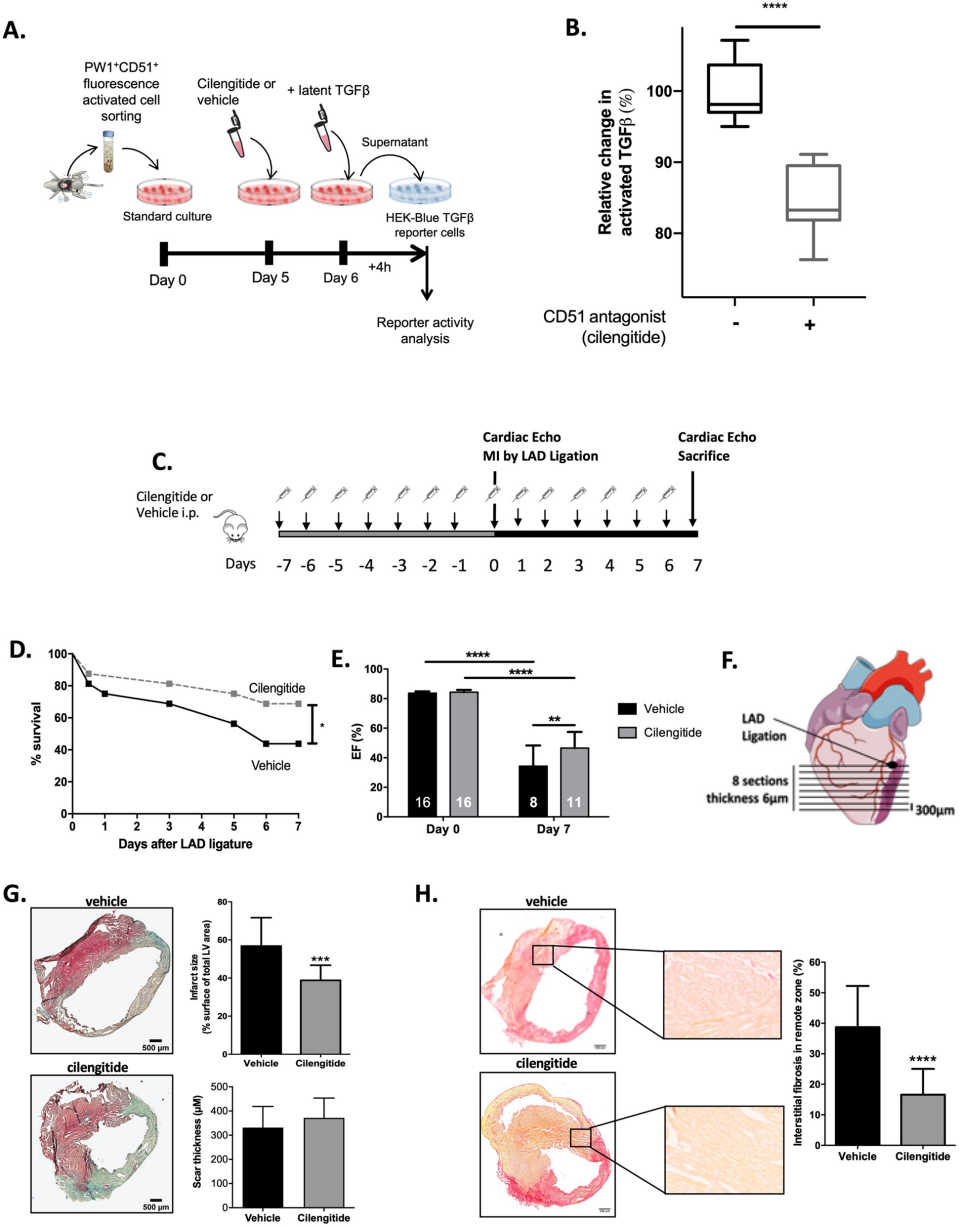
Cilengitide reduces cardiac fibrosis and improves cardiac function after MI. (**A**) Design of the in vitro experiments to test the ability of isolated PW1^+^CD51^+^ cardiac cells to activate latent TGFβ. (**B**) TGFβ levels in isolated PW1^+^CD51^+^ cardiac cells supplemented with latent TGFβ and treated with or without the anti-integrin drug cilengitide (10 µM final concentration). Results are normalized to absolute values measured in vehicle-treated group. N = 9 from three independent isolates. ****P < 0.0001 using Mann–Whitney test between groups. (**C**) Experimental design: Mice were pre-treated for 7 days with cilengitide or vehicle (N = 16 per groups) before MI induction (Day 0) and then treated for 7 additional days. (**D**) Survival curves of cilengitide- and vehicle-treated mice 7 days after MI. *P < 0.05, log-rank test. (**E**) Left ventricular ejection fractions of cilengitide- and vehicle-treated mice immediately before surgery and 7 days later. **P < 0.01, ****P < 0.0001. (**F**) Analysis of infarct size and cardiac fibrosis between cilengitide- and vehicle-treated mice 7 days after MI was performed at 8 levels below the LAD ligation site. (**G**) Masson trichome staining in vehicle- and cilengitide-treated animals and quantification of infarct size and scar thickness between both groups, ***P < 0.001 (N = 4 per group). (**H**) Sirius red staining in vehicle- and cilengitide-treated animals and quantification of interstitial fibrosis in remote areas between both groups, ****P < 0.0001, (N = 4 per group). Data are mean ± SD. Drawings are from servier medical art (https://smart.servier.com) which is licenced under a creative commons attribution 3.0 unported license.

We first FACS-isolated and cultured cardiac PW1^+^CD51^+^ cells, which were then incubated with latent TGFβ in presence or absence of the anti-integrin drug cilengitide for 24 h. Using HEK-Blue TGFβ reporter cells that allow the detection of bioactive TGFβ by monitoring the activation of the TGFβ/Smad pathway (Fig. 5A), we found a significant reduction in the levels of active TGFβ in the supernatants of PW1^+^CD51^+^ cells treated with cilengitide as compared to vehicle (Fig. 5B). This observation highlights the direct contribution of PW1^+^CD51^+^ cells in the activation of the fibrosis mediator TGFβ.

In addition, cilengitide treatment affected the expression of profibrotic genes in cultured cardiac PW1^+^ cells, as evident from the significant downregulation in the levels of *Acta2*, *Mmp2*, and *Tgfrb1* (SI Fig. S7). This result is suggestive of the reduction in TGFβ signaling and myofibroblast differentiation capacity. *Col1a1* expression was, however, unaffected by cilengitide treatment, supporting the specific regulatory role on individual ECM genes.

### Targeting PW1^+^ αV-integrin reduce cardiac fibrosis after MI

We examined the potential of the pharmacological blockade of αV-integrin to prevent cardiac fibrosis in response to ischemic injury. Mice were pre-treated for 7 days with cilengitide or vehicle and then subjected to MI via a complete LAD ligation. Mice were daily treated with cilengitide or vehicle for 7 additional days before the final evaluation of cardiac function and remodeling (Fig. 5C). We found that cilengitide treatment significantly improved the animal survival (Fig. 5D) and significantly increased their cardiac functions, as analyzed by left ventricular ejection fraction (Fig. 5E). Staining for collagen (Masson trichrome and Sirius red) was performed on eight sequential cardiac sections (Fig. 5F). Digital image quantification demonstrated a significant reduction in the infarct size (38.8% ± 7.9% versus 56.8% ± 14.9% in cilengitide versus vehicle, P = 0.0008, Fig. 5G) and interstitial fibrosis measured in the remote myocardial area (15.9% ± 10.4% versus 36.8% ± 13.1% in cilengitide versus vehicle, P < 0.0001, Fig. 5H) following cilengitide treatment, which was not associated with any changes in survival, cardiac function, or cardiac fibrosis in sham animals (SI Fig. S8). There were no significant differences in scar thickness between groups (Fig. 5G).

**Figure 6:**
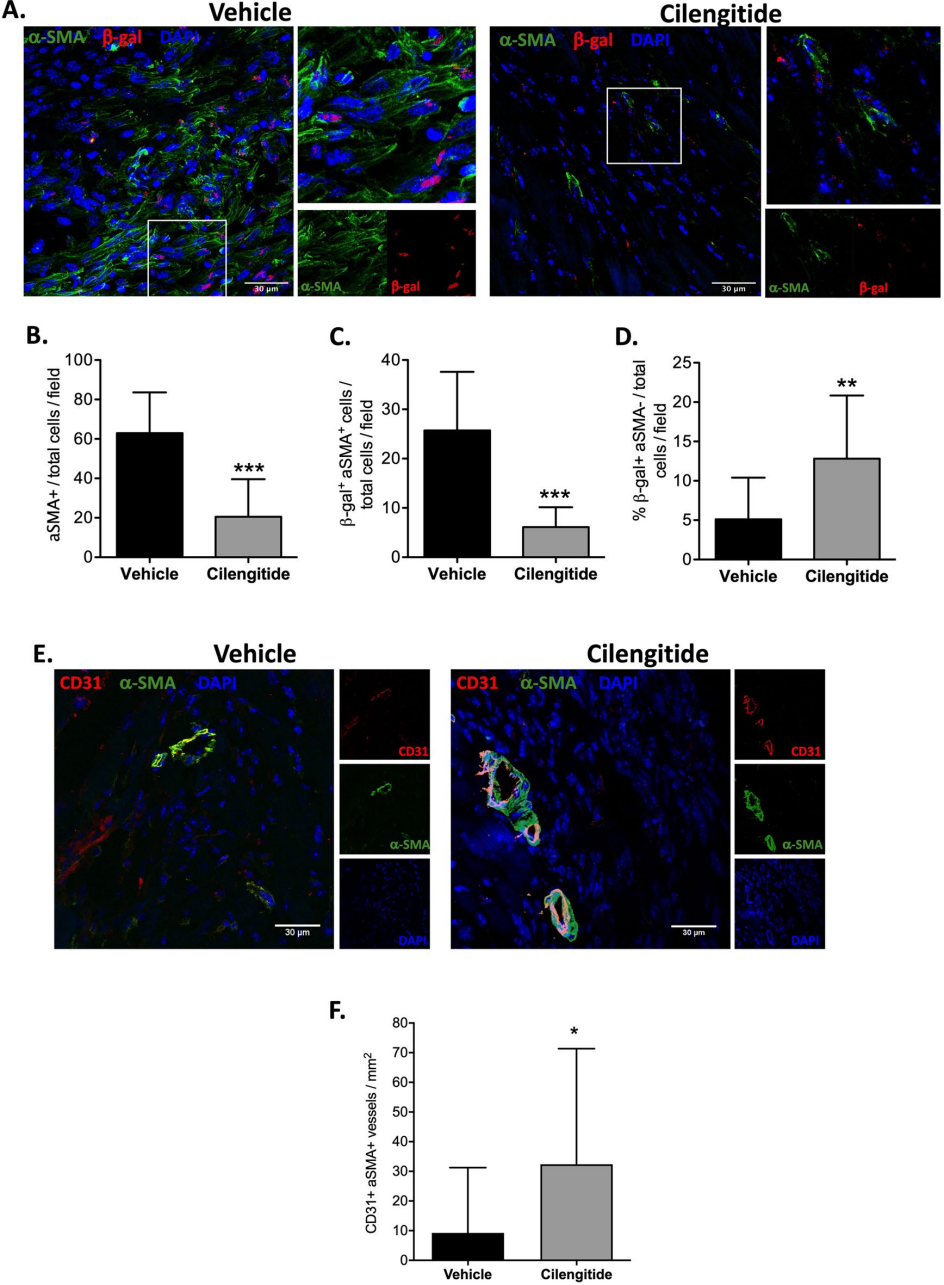
Cilengitide reduces the profibrotic activation of cardiac PW1^+^ cells. (**A**) Representative cardiac sections (in the infarct zone) from vehicle- and cilengitide-treated mouse hearts immunostained with αSMA (green), β-gal (red), and DAPI (blue). White arrows indicate β-gal single-positive cells. (**B**) Quantification of αSMA^+^ cells within the total number of cells per field in vehicle- and cilengitide-treated post-MI hearts. (**C**) Quantification of β-gal^+^αSMA^+^ cells within the total number of cells per field in vehicle- and cilengitide-treated post-MI hearts. (**D**) Quantification of β-gal^+^ cells within the αSMA-negative cells in vehicle- and cilengitide-treated post-MI heart sections. (**E**) Representative cardiac sections (in the border zone) from vehicle- and cilengitide-treated mouse hearts immunostained with αSMA (green), CD31 (red), and DAPI (blue). (**F**) Quantification of CD31^+^αSMA^+^ vessels in the border zone in vehicle- and cilengitide-treated post-MI hearts. N = 18 fields at least from two animals per groups, *P < 0.05, **P < 0.01 and ***P < 0.001. Data are mean ± SD.

As these results suggest a reduction in the mechanisms leading to the development of cardiac fibrosis in response to MI, we used the stability (produrance) of the β-gal reporter to perform short-term lineage tracing of cells derived from β-gal^+^ cells (i.e., cardiac PW1^+^ cells) using the PW1^nLacZ^-reporter mouse model. We examined β-gal activity and expression of αSMA as a marker of activated fibroblasts in vivo in post-MI (7 days) heart sections. As a result, we found that cilengitide treatment significantly reduced the total number of αSMA^+^ cells (Fig. 6A,B) and the proportion of cells co-expressing β-gal and αSMA (Fig. 6C) and significantly increased the number of β-gal^+^ cells lacking αSMA expression (Fig. 6D). In addition, we also found a significantly higher number of CD31^+^αSMA^+^ vessels in the border zone in cilengitide-treated animals as compared to vehicle (Fig. 6E,F). Overall, these results suggest that αV-integrin blockade with cilengitide affects the ability of cardiac PW1^+^ cells to differentiate into fibroblasts and contribute to fibrotic scarring and contributes to the preservation of vasculature in the border areas.

## Discussion

To the best of our knowledge, here we provide the first description of the proteins expressed on the surface of cardiac PW1^+^ cells and identify a novel target to suppress the fibrogenic behavior of these cells, resulting in favorable outcomes in post-MI cardiac remodeling and animal survival.

The transgenic PW1 reporter mouse model has facilitated the identification of PW1^+^ cells and demonstrated the importance of PW1 as a pan-tissue stem cell marker^19,20,26–28^. Herein, we used this model to specifically isolate cardiac PW1^+^ cells and adopt a multi-omic unbiased approach to further decipher the cell surface membranome of these cells. The transcriptomic predictions allowed filtering of a large number of membrane proteins identified through MS technique and, hence, increased the likelihood of identification of a more specific cell surface marker among the limited number of shortlisted candidates. A similar approach was recently proposed to define the secretome of bone marrow stem cells^29^. Here, we have further expanded this approach by adding a proteome membrane expression profiling in order to better specify candidates among the large set of candidates generated by the transcriptomic predictions. The overlap between the transcriptomic and proteomic datasets led to the selection of more specific and relevant candidates.

We found αV-integrin (CD51) is expressed on the majority of cardiac PW1^+^ cells which was not previously reported or anticipated. Data on the expression of CD51 in cardiac cells are limited and CD51 was previously reported to be expressed on > 90% of cardiac fibroblasts cultivated in vitro for 5 days after isolation^30^. RNA-seq of the freshly isolated cells revealed the positive expression of CD51 on freshly isolated cardiac PW1^+^ cells, which exhibited a fibroblast-like gene signature. Single-cell RNA-seq analysis showed that both markers did not specifically tag a single cell population but were mostly expressed in fibroblasts, further supporting the notion that cardiac PW1^+^CD51^+^ cells comprise a subpopulation within the heterogeneous population of cells commonly referred as cardiac fibroblasts. Reciprocally, αV-integrin (CD51) expression was predominantly detected on cells expressing PW1^+^ in the myocardium, thereby highlighting the discriminative value of this new marker that could henceforth be useful for PW1^+^ cell sorting. Whether αV-integrin (CD51) specifically tags PW1^+^ cells in non-cardiac organs, however, remains to be determined.

The identification of this new cell surface marker provides important information on the pathophysiological role of cardiac PW1^+^ cells. CD51 is an αV-integrin subunit, a subset of the integrin family cell adhesion receptors that was thought to serve as a central mediator of organ fibrosis through TGFβ activation^22,23^. Our results indicate that αV-integrin expressed by cardiac PW1^+^ cells activate latent TGFβ and that the pharmacological blockade of αV-integrin could reduce TGFβ activation in vitro and cardiac fibrosis post-MI in vivo. This new observation is consistent with our previous findings that cardiac PW1^+^ cells display a fibrogenic behavior in response to ischemic injury, notably by directly giving rise to fibroblasts^19^*.* However, the pharmacological blockade of αV-integrin could suppress the expression of fibrotic genes in isolated cardiac PW1^+^ cells and reduce their ability to differentiate into fibroblasts in vivo post-MI, suggestive of the role of αV-integrin as a regulator of multiple pro-fibrotic mechanisms. Overall, our results are compatible with the hypothesis that cardiac PW1^+^ cells represent a source of fibrosis by directly differentiating into fibroblasts as well as by orchestrating a microenvironment that further favors the activation of resident fibroblasts or remodeling of the ECM.

Pharmacological blockade of αV-integrin with cilengitide in this model significantly reduced post-MI fibrotic remodeling, consistent with the significant reduction in infarct size and interstitial fibrosis but without any increase in the rate of cardiac rupture. The survival and cardiac function of cilengitide-treated animals consequently improved. Our study thus suggests the potential development of a new anti-fibrotic therapy by targeting αV-integrin expressed on fibrogenic cardiac PW1^+^ cells. So far, HF therapies are directed to alleviate cardiac workload and indirectly reduce cardiac remodeling; however, there is no effective anti-fibrotic therapeutic strategy that could complement the current therapies for HF^9,11,12^. Anti-fibrotic strategies in MI are often associated with the risk of cardiac rupture by limiting the development of replacement fibrosis, which restores the region devoid of viable CMs and prevents cardiac rupture^31^. For instance, it was recently shown that strategies that limit inflammatory cell recruitment to the site of infarction delayed the removal of dead CMs and their replacement by a scar tissue, consequently increasing the susceptibility to cardiac rupture^32^. We failed to observe such an adverse outcome, instead noticed significant improvements in mouse survival after cilengitide treatment. The significant reduction in interstitial fibrosis in cilengitide-treated animals suggests that the blockade of αV-integrin does not directly affect the replacement fibrotic remodeling but rather limits the development of reactive interstitial fibrosis in the viable myocardium^12^. The role of integrins in non-cardiac organ fibrosis (i.e., lung and liver) is being increasingly recognized, and genetic deletion or pharmacological inhibition of integrin was shown to be associated with reduced fibrotic remodeling^23,33,34^. Interestingly, these recent studies indicate the critical role of αVβ1 in organ fibrosis^23,33^. Our data show co-expression of αV-integrin and ITGβ1 in cardiac PW1^+^ cells although we do not provide evidence that these molecules are dimerized. Indeed. the roles of αVβ1 heterodimers as well as mesenchymal-like cells αV integrins in the development of muscular and cardiac fibrosis have only been recently reported^23,35^. We therefore provide evidence for the role of cardiac PW1^+^ αV-integrin cells in the development of cardiac fibrosis. Additional experiments are needed to decipher the expression and functional role of αV-heterodimers within the different populations of cardiac cells.

Our study has some limitations that should be acknowledged. First, integrins are expressed on a variety of cells. αV-integrin is expressed on endothelial cells and cilengitide is thought to exert some anti-angiogenic effects^36^. It is unlikely that the observations reported herein could be attributed to the anti-angiogenic effects of cilengitide, as these effects would rather contribute to the adverse deterioration of cardiac function and remodeling post-MI. We rather found an increase in small vessel in the border zone of cilengitide-treated animals, suggesting a beneficial effect on vasculature. ITGβ3 is similarly expressed by endothelial cells; however, we observed a significant increase in ITGβ3 expression level in the infarct area, a region typically depleted of vessels but enriched with PW1^+^ cells. Overall, this result suggests that the observed benefit with cardiac fibrotic remodeling is primarily associated with the reduction in the fibrogenic behavior of cardiac PW1^+^ cells and to a potential benefit on the vascular compartment. The contribution of circulating cells (such as inflammatory cells in response to CM necrosis) may however not be ruled out. Similarly, we did not explore the effect of cilengitide on immune and inflammatory responses after MI, but there is currently no evidence of an action of cilengitide on immune cells. This issue would deserve further investigations. In addition, we did not explore whether PW1, as a transcription factor, directly controls the expression of fibrotic genes. Constitutive Peg3/PW1 knock-out mice were recently generated and showed reduction in post-natal growth, supporting the direct role of PW1 in the regulation of tissue growth^37^. Further experiments with inducible-knockout mouse models would be warranted to address this question. Importantly, we cannot rule out a contribution of other cell types as αV-integrin expression was also observed on a small proportion of cells which do not express PW1 (Fig. 3D,E).

Our results provide a route to translate towards a clinical setting as anti-CD51 antibodies (namely abituzumab and intetumumab) and small molecules have recently been developed and have entered into phase 1/2 clinical trials. These molecules were developed to primarily block cell proliferation, adhesion, and migration in relation to the function of αV-integrin and have thus been tested in patients with cancer, although with modest efficacy^38^. Our study indicates that these molecules could be repurposed for the treatment of fibrotic disorders, including post-MI cardiac fibrosis.

## Conclusions

Overall, these data identify cardiac PW1^+^ cells as a source of cardiac fibrosis in response to ischemic injury via αV-integrin and suggest that the pharmacological targeting of αV-integrin may offer clinical benefits in the treatment of cardiac ischemia.

## Methods

Additional description of experiments is provided in the supplemental material appendix.

All procedures and animal care protocols were approved by our institutional research committee (CEEA34 and French ministry of research) and conformed to the animal care guideline in Directive 2010/63/EU European Parliament. All animals received humane care in compliance with the “Principles of Laboratory Animal Care” formulated by the National Society for Medical Research and the “Guide for the Care and Use of Laboratory Animals” prepared by the Institute of Laboratory Animal Resources and published by the National Institutes of Health (NIH Publication No. 86-23, revised 1996).

### Study design

Left anterior descending artery (LAD) surgery was performed on 8-week-old male C57BL/6J and PW1-reporter (PW1^nLacZ^) mice, which were anesthetized in an induction chamber with 2% isoflurane mixed with 1.0 L/min 100% O_2_ and placed in a supine position on a heating pad to maintain body temperature. The mice were intubated with endotracheal tube and then connected to a rodent ventilator (180 breaths/min and a tidal volume of 200 µL). During surgical procedure, anesthesia was maintained at 1.5–2% isoflurane with O_2._ The chest was accessed from the left side through the intercostal space, and the pericardium incised. The LAD was exposed and encircled with an 8-0 prolene suture at the proximal position. The suture was briefly snared to confirm the ligation by blanching the arterial region. For in vivo pharmacological studies, cilengitide (10 mg/kg; AdooQ Bioscience) was daily intraperitoneally administered to mice 7 days before and then 7 days after MI in line with previous reports^39^. Mice from the control group were treated with vehicle. At day 7 after surgery, animals were euthanatized and hearts were carefully excised, fixed in paraformaldehyde 4% and then incubated with 30% sucrose solution overnight. Hearts were immersed in optimum cutting temperature (OCT) embedding medium and immediately frozen in liquid nitrogen vapors.

### Measurement of cardiac parameters

Echocardiographic measurements were performed at day 0 and 7 after surgery to assess cardiac function using two-dimensionally guided Time Motion mode recording (Vivid7 PRO apparatus, GE Medical System Co). Each set of measurements was obtained from the same cardiac cycle.

### Cell isolation and fluorescence-activated cell sorting (FACS)

Cells were sorted from the whole heart (upon atria removal) of 8-week-old PW1^nLacZ^ mice as previously described^19^. Briefly, after mouse euthanasia, hearts were perfused with phosphate-buffered saline (PBS) to flush out any residual blood cells. Ventricles were finely minced (0.5–1 mm^3^) and then enzymatically digested for 30 min at 37 °C with a 480 U/mL collagenase II (Worthington) solution in PBS. Dulbecco’s modified Eagle’s medium (DMEM, Life Technologies) supplemented with 10% fetal bovine serum (FBS, Sigma) and 1% penicillin/streptomycin (P/S) (Life Technologies) was added to terminate digestion, and the obtained cell suspension was filtered through a 100 µm cell strainer before centrifugation at 400×*g* and 4 °C for 10 min. Red blood cell lysing (RBCL) buffer was added to the cell pellet to lyse red blood cells. The reaction was terminated with Hanks’ Balanced Salt solution (Gibco) containing 1% FBS (HBSS-1% FBS), followed by centrifugation under same conditions as above. Cell suspension was then stained on ice for 45 min in the dark with several antibodies, listed in SI Table S1, diluted in HBSS-1% FBS. Cells were rinsed with HBSS-1% FBS and centrifuged before staining for PW1.

To detect β-gal reporter activity (PW1^+^ cells), cells were incubated in the dark with HBSS-1% FBS containing 60 µM of the β-gal substrate 5-dodecanoylaminofluorescein di-β-d-galactopyranoside (C12FDG, Thermo Fisher Scientific) at 37 °C for 1 h under constant agitation. HBSS-1% FBS was added to stop the reaction. After centrifugation, the samples were incubated with propidium iodide (Sigma) and then filtered using a 50 µm Filcon (BD bioscience) before analysis. The different populations were gated, analyzed, and sorted on a FACS Aria II cytometer (BD Biosciences). Viability threshold was determined by comparing conditions with and without PI. All immunostained populations were obtained by comparing signals from unstained cells and fluorescence minus one conditions. β-Gal activity threshold was obtained by comparing β-gal-negative and -positive cells.

### RNA sequencing (RNA-seq)

In total, 300 ng of total RNA extracted from freshly isolated cells with SureSelect Strand-Specific RNA kit (Agilent) was used to prepare a library, according to the manufacturer’s instructions. The resulting library was quality checked and quantified by peak integration on Bioanalyzer High sensitivity DNA labchip (Agilent). A pool of equal quantity of 12 purified libraries was prepared, and each library was tagged with a different index. mRNAs pool libraries were finally sequenced on Illumina Hiseq 1500 instrument using a rapid flowcell. The pool was loaded on two lanes of the flowcell. A paired-end sequencing of 2 × 100 bp was performed.

After discarding reads that did not pass the Illumina filters and trimming low-quality sequenced bases (q < 28) using the Cutadapt program^40^, we restricted our downstream analyses to reads with lengths greater than 90 bp. Selected reads were mapped to a murine reference transcriptome that was generated by the RSEM package^41^ from the full mouse reference genome and the gtf transcript annotations file from ENSEMBL^42^. Alignment and estimation of transcripts abundance in each of the 12 processed samples were performed using the RSEM program. Transcripts with abundance counts higher than 10 in more than two samples (N = 36,948) were considered as expressed and retained for further analysis. Abundances of transcripts assigned to the same gene were combined together, leading to the profiling of 16,403 genes. Analyses were conducted under the R environment (version 3.2.2).

Galaxy 15.10 instance was locally installed on a server machine. WolfPsort, TMHMM, and SignalP were obtained from CBS prediction servers (https://services.healthtech.dtu.dk/, accessed April 15th, 2020). NLStradamus and PredictNLS were used in parallel to determine nuclear localization signals. Each dataset from RNA-seq, corresponding to a different population, was then processed through a pipeline designed to select sequences containing a signal peptide, at least one transmembrane segment, no nuclear localization signal, and a theoretical presence at the plasma membrane. All samples were compared, and a list of candidates that were only expressed in PW1^+^ cells under normal and post-MI conditions was obtained.

### Single-cell RNA-seq

Single cell RNA-seq data were obtained from a previously published study of cardiac non-myocytes isolated from left ventricles of 10-week-old mice at homeostasis^25^. Cell-type labels for each cell profiled in this previous study are available at the following URL: https://github.com/daskelly/CellReports_2018_heart_scRNAseq. Data were analyzed and plots were constructed with the Seurat package version 2.3.4^43^ using the R environment for statistical computing version 3.5.0^44^.

### PW1^+^CD51^+^ cell culture and TGFβ supernatant assay

FACS-sorted cells were seeded at a density of 25,000 cells per cm^2^ in culture plates coated with attachment factor (Thermo Fisher Scientific) in DMEM supplemented with 10% FBS and 1% P/S. The culture medium was changed the day after and treatments were performed on day 5.

For the analysis of the activation of latent transforming growth factor beta (TGFβ), PW1^+^CD51^+^ cells were incubated in a medium supplemented with latent TGFβ (1.1 ng/mL final concentration) in the presence of cilengitide (10 μM final concentration) or vehicle for 24 h at 37 °C. HEK-Blue TGFβ cells (InvivoGen) were seeded in 24-well plates at a density of 20,000 cells/well in the same medium as PW1^+^CD51^+^ cells. Following attachment, these cells were incubated with the supernatant from PW1^+^CD51^+^ cells treated with latent TGFβ and cilengitide or vehicle. Internal positive controls included HEK-Blue cells treated with active TGFβ (3 ng/mL final concentration), while the negative control was HEK-Blue cells treated with latent TGFβ (1.1 ng/mL) in medium. Following overnight incubation, 100 μL of medium from cells under each treatment condition was collected and incubated with 160 μL of Quanti-Blue medium in 96-well plates at 37 °C for 2 h. The absorbance was measured at 625 nm wavelength using a plate reader (FlexStation 3, Molecular Devices).

For PW1^+^ cell transcriptional signature analysis after cilengitide treatement, PW1^+^ cells were stimulated with 300 and 1,000 nM of cilengitide. At day 6, medium was removed and cells were washed twice with PBS before RNA extraction following manufacturer’s protocol (PureLink™ RNA mini kit, Life Technologies). RNA was converted to cDNA using the SuperScript IV First-Strand Synthesis System (Thermo Fisher Scientific) to perform quantitative polymerase chain reaction (q-PCR) (Sybr Select Master Mix, Life Technologies) for the analysis of expression of selected fibrosis markers. The list of primers used is reported in SI Table S2.

### Statistical analysis

The data are expressed as mean ± standard deviation (SD). Quantitative data were analyzed using one-way analysis of variance (ANOVA) and pair-wise comparisons with Tukey’s test for multiple comparisons. The Mann–Whitney U test was used for comparing continuous variables between two groups. A log-rank (Mantel–Cox) test was employed to compare survival between groups. A value of P < 0.05 was considered significant. All analyses were performed by using GraphPad Prism 7.

## Supplementary information

Supplementary file1 (DOCX 7075 kb)

## References

[1] Maggioni AP (2013). EURObservational Research Programme: Regional differences and 1-year follow-up results of the Heart Failure Pilot Survey (ESC-HF Pilot). Eur. J. Heart. Fail..

[2] Roger VL (2012). Heart disease and stroke statistics—2012 update: A report from the American Heart Association. Circulation.

[3] Seferovic PM (2013). Organization of heart failure management in European Society of Cardiology member countries: survey of the Heart Failure Association of the European Society of Cardiology in collaboration with the Heart Failure National Societies/Working Groups. Eur. J. Heart. Fail..

[4] Ponikowski P (2016). 2016 ESC Guidelines for the diagnosis and treatment of acute and chronic heart failure: The Task Force for the diagnosis and treatment of acute and chronic heart failure of the European Society of Cardiology (ESC) Developed with the special contribution of the Heart Failure Association (HFA) of the ESC. Eur. Heart J..

[5] Savarese G, Lund LH (2017). Global public health burden of heart failure. Cardiac. Fail. Rev..

[6] Dunlay SM, Roger VL, Redfield MM (2017). Epidemiology of heart failure with preserved ejection fraction. Nat. Rev. Cardiol..

[7] Lewis GA (2017). Biological Phenotypes of Heart Failure With Preserved Ejection Fraction. J Am Coll Cardiol.

[8] Gyongyosi M (2017). Myocardial fibrosis: biomedical research from bench to bedside. Eur. J. Heart. Fail..

[9] Schelbert EB, Fonarow GC, Bonow RO, Butler J, Gheorghiade M (2014). Therapeutic targets in heart failure: Refocusing on the myocardial interstitium. J. Am. Coll. Cardiol..

[10] Li AH, Liu PP, Villarreal FJ, Garcia RA (2014). Dynamic changes in myocardial matrix and relevance to disease: Translational perspectives. Circ. Res..

[11] Gourdie RG, Dimmeler S, Kohl P (2016). Novel therapeutic strategies targeting fibroblasts and fibrosis in heart disease. Nat. Rev. Drug Discov..

[12] Travers JG, Kamal FA, Robbins J, Yutzey KE, Blaxall BC (2016). Cardiac fibrosis: The fibroblast awakens. Circ. Res..

[13] Kramann R (2015). Perivascular Gli1+ progenitors are key contributors to injury-induced organ fibrosis. Cell Stem Cell.

[14] Kalluri R (2016). The biology and function of fibroblasts in cancer. Nat. Rev. Cancer.

[15] Teekakirikul P (2010). Cardiac fibrosis in mice with hypertrophic cardiomyopathy is mediated by non-myocyte proliferation and requires Tgf-beta. J. Clin. Invest..

[16] Olivey HE, Mundell NA, Austin AF, Barnett JV (2006). Transforming growth factor-beta stimulates epithelial–mesenchymal transformation in the proepicardium. Dev. Dyn..

[17] Duan J (2012). Wnt1/betacatenin injury response activates the epicardium and cardiac fibroblasts to promote cardiac repair. EMBO J..

[18] Humphreys BD (2010). Fate tracing reveals the pericyte and not epithelial origin of myofibroblasts in kidney fibrosis. Am. J. Pathol..

[19] Yaniz-Galende E (2017). Fibrogenic potential of PW1/Peg3 expressing cardiac stem cells. J. Am. Coll. Cardiol..

[20] Besson V (2011). PW1 gene/paternally expressed gene 3 (PW1/Peg3) identifies multiple adult stem and progenitor cell populations. Proc. Natl. Acad. Sci. USA.

[21] Mitchell KJ (2010). Identification and characterization of a non-satellite cell muscle resident progenitor during postnatal development. Nat. Cell Biol..

[22] Henderson NC (2013). Targeting of alphav integrin identifies a core molecular pathway that regulates fibrosis in several organs. Nat. Med..

[23] Chen C, Li R, Ross RS, Manso AM (2016). Integrins and integrin-related proteins in cardiac fibrosis. J. Mol. Cell. Cardiol..

[24] Salven P, Mustjoki S, Alitalo R, Alitalo K, Rafii S (2003). VEGFR-3 and CD133 identify a population of CD34+ lymphatic/vascular endothelial precursor cells. Blood.

[25] Skelly DA (2018). Single-cell transcriptional profiling reveals cellular diversity and intercommunication in the mouse heart. Cell Rep..

[26] Dierick F (2016). Resident PW1+ progenitor cells participate in vascular remodeling during pulmonary arterial hypertension. Circ. Res..

[27] Sojoodi M (2016). The zinc finger transcription factor PW1/PEG3 restrains murine beta cell cycling. Diabetologia.

[28] Besson V (2017). Expression analysis of the stem cell marker Pw1/Peg3 reveals a CD34 negative progenitor population in the hair follicle. Stem Cells.

[29] Korf-Klingebiel M (2015). Myeloid-derived growth factor (C19orf10) mediates cardiac repair following myocardial infarction. Nat. Med..

[30] Furtado MB (2014). Cardiogenic genes expressed in cardiac fibroblasts contribute to heart development and repair. Circ. Res..

[31] Sun Y, Weber KT (2000). Infarct scar: A dynamic tissue. Cardiovasc. Res..

[32] Lorchner H (2015). Myocardial healing requires Reg3beta-dependent accumulation of macrophages in the ischemic heart. Nat. Med..

[33] Reed NI (2015). The alphavbeta1 integrin plays a critical in vivo role in tissue fibrosis. Sci. Transl. Med..

[34] Conroy KP, Kitto LJ, Henderson NC (2016). alphav integrins: key regulators of tissue fibrosis. Cell Tissue Res..

[35] Murray IR (2017). Alphav integrins on mesenchymal cells regulate skeletal and cardiac muscle fibrosis. Nat. Commun..

[36] Nisato RE, Tille JC, Jonczyk A, Goodman SL, Pepper MS (2003). alphav beta 3 and alphav beta 5 integrin antagonists inhibit angiogenesis in vitro. Angiogenesis.

[37] Denizot AL (2016). A novel mutant allele of Pw1/Peg3 does not affect maternal behavior or nursing behavior. PLoS Genet..

[38] Lasinska I, Mackiewicz J (2018). Integrins as a new target for cancer treatment. Anticancer Agents Med. Chem..

[39] Mas-Moruno C, Rechenmacher F, Kessler H (2010). Cilengitide: the first anti-angiogenic small molecule drug candidate design, synthesis and clinical evaluation. Anticancer Agents Med. Chem..

[40] Chen C, Khaleel SS, Huang H, Wu CH (2014). Software for pre-processing Illumina next-generation sequencing short read sequences. Source Code Biol. Med..

[41] Li B, Dewey CN (2011). RSEM: Accurate transcript quantification from RNA-Seq data with or without a reference genome. BMC Bioinform..

[42] Yates A (2016). Ensembl 2016. Nucleic Acids Res..

[43] Butler A, Hoffman P, Smibert P, Papalexi E, Satija R (2018). Integrating single-cell transcriptomic data across different conditions, technologies, and species. Nat. Biotechnol..

[44] R Foundation for Statistical Computing (2018). A Language and Environment for Statistical Computing.

