# Supplementary material for: Anti-integrin αv therapy improves cardiac fibrosis after myocardial infarction by blunting cardiac PW1+ stromal cells
Source: Sci Rep. 2020 Jul 9;10:11404. doi: 10.1038/s41598-020-68223-8 (PMC7347632; doi:10.1038/s41598-020-68223-8)
Supplement: Supplementary file 1 — Supplementary file1 (DOCX 7075 kb) [file 41598_2020_68223_MOESM1_ESM.docx]

**ANTI-INTEGRIN α_v_ THERAPY IMPROVES CARDIAC FIBROSIS AFTER MYOCARDIAL INFARCTION BY BLUNTING CARDIAC PW1 ^+^ STROMAL CELLS**

Marion Bouvet^1†^ PhD, Olivier Claude^1†^ PhD, Maguelonne Roux^2^ PhD, Dan Skelly^3^ PhD, Nihar Masurkar PhD^1^, Nathalie Mougenot^4^ PhD, Sophie Nadaud^2^ PhD, Catherine Blanc^5^ PhD, Clément Delacroix^1^ PhD, Solenne Chardonnet^5^ PhD, Cédric Pionneau^5^ PhD, Claire Perret^2^ MSc, Elisa Yaniz-Galende^2^ PhD, Nadia Rosenthal^3^ MD PhD, David-Alexandre Trégouët^2,6^ PhD, Giovanna Marazzi^1^ MD PhD, Jean-Sébastien Silvestre^1^ PhD, David Sassoon^1^ PhD and Jean-Sébastien Hulot^1*^ MD PhD

**Affiliations:**

1. Université de Paris, PARCC, INSERM, F-75015 Paris, France.
2. Sorbonne Université, UPMC Univ Paris 06, INSERM, Institute of Cardio metabolism and Nutrition (ICAN), Paris, France.
3. The Jackson Laboratory, Bar Harbor, ME, USA
4. Sorbonne Université, UPMC Univ Paris 06, PECMV, UMS28, Paris, France.
5. Sorbonne Université, Inserm, UMS Omique, Plateforme Post-génomique de la Pitié-Salpêtrière, P3S, F-75013, Paris, France
6. INSERM UMR_S 1219, Bordeaux Population Health Research Center, University of Bordeaux, France

**SUPPLEMENTARY INFORMATION**

**Supplementary materials and methods**

**Supplementary figures: 8**

**Supplementary tables: 2**

**Supplementary materials and methods:**

**Proteomics**

Proteins from 300,000 PW1^+^ sorted cells were extracted with Dounce-Potter homogenization into an ice-cold lysis buffer (10 mM HEPES, 1.5 mM magnesium chloride, 10 mM potassium chloride, 0.5 mM dithiothreitol [DTT], 1 mM orthovanadate, and protein inhibitor cocktail). The homogenate was centrifuged at 600 ×*g* for 10 min at 4°C, and the obtained supernatant was further centrifuged for 25 min at 100,000 ×*g* and 4°C. The pellet was rinsed (50 mM Tris, 0.15 mM phenylmethylsulfonyl fluoride, and protein inhibitor cocktail) and centrifuged as mentioned above. The final pellet was homogenized in 50 mM ammonium bicarbonate [NH_4_HCO_3_]. Membranous proteins were separated on sodium dodecyl sulfate polyacrylamide gel electrophoresis (SDS-PAGE) gels (12%) and stained as per a MS-compliant silver nitrate staining protocol. All proteins were excised from one large strip, which was minced into 1 mm^3^ cubes and subjected to protein reduction, alkylation (DTT, iodoacetamide), and trypsinization (500 ng in 50 mM NH_4_HCO_3_, acetonitrile 5%). Finally, peptides were extracted with a 20 µL solution of 60% acetonitrile and 0.1% formic acid, dried in a speed-vacuum, and resuspended in a 20 µL solution of 3% acetonitrile and 0.1% formic acid. Each sample (4 µL) was analyzed by liquid chromatography tandem mass spectrometry (LC-MS/MS, Ultimate 3000 Rapid Separation liquid chromatographic system coupled to a hybrid Q Exactive Plus mass spectrometer, Thermo Fisher Scientific). Briefly, peptides were loaded onto a C18 reverse phase precolumn (3 µm particle size, 100 Å pore size, 75 µm i.d., 2 cm length) in 0.1% trifluoroacetic acid and 2% acetonitrile. Peptides were then separated on a C18 reverse phase resin (2 µm particle size, 100 Å pore size, 75 µm i.d., 15 cm length) with a gradient from 98% solvent A (0.1% formic acid in high-performance liquid chromatography [HPLC]-grade water) to 45% solvent B (80% acetonitrile and 0.085% formic acid in HPLC-grade water) for 97 min. The Q Exactive Plus mass spectrometer acquired data throughout the elution process and operated in a data-dependent scheme with full MS scans acquired with the orbitrap, followed by stepped higher energy collisional dissociation on the most abundant ions detected in the MS scan. Mass spectrometer settings were as follows: full MS (AGC: 3E6, resolution: 7E4, m/z range 375-1500, maximum ion injection time: 100 ms); MS/MS (Normalized Collision Energy: 30, resolution: 17500, intensity threshold: 1E4, isolation window: 4.0 m/z, dynamic exclusion time setting: 30 s, AGC Target: 1E5 and maximum injection time: 100 ms). Fragmentation was permitted for precursors with a charge state of 2 to 4. Proteome discoverer 1.4 was used to generate .mgf files. Protein identification was performed using X!Tandem (Ver: 2015.04.01.1) with X!TandemPipeline (Ver: 3.4.3) (37). Analysis was performed with UniProt mouse reference proteome database downloaded on Dec. 12, 2016, and with a standard contaminant database using the following parameters: 1 missed cleavage, carbamidomethylation of Cys as a fixed modification, acetylation of protein N-ter and oxidation of Met as variable modifications, MS tolerance at 5 ppm and MS/MS tolerance at 20 ppm and possibility of semi-tryptic peptides. At least 1 peptide with a p value < 0.05 and a protein score Log(e value) < −2 was required for protein validation. With these parameters, the false discovery rate (FDR) of peptide was 0.128 and that of protein was 0.95. Final results provided 1,679 groups with 1,831 subgroups. The protein list obtained from LC-MS analysis has been manually filtered to retain only plasma membrane proteins.

**Histological analysis**

OCT-embedded heart blocks were cutted using a cryostat (Leica CM 3050). Cryosections (6 µm thickness) were collected (every 200-300 µm) from the apex to the base of the heart. Briefly, for Sirus red staining, heart sections were hydrated before 2 min incubation in 0.2% phosphomolybdic acid solution and then 1 h staining in picro-sirius red solution (0.1% [m/v] Sirus Red F3B (CI 35780) in saturated aqueous solution of picric acid (1.3% in water)). After staining, heart sections were washed for 2 min in a 0.01N hydrochloric acid solution and then dehydrated by sequential transfer in 75%, 95% and 100% ethanol solutions. The sections were then cleared in xylene and mounted in a resinous medium (Eukitt quick-hardening mounting-medium, Sigma). For Masson’s trichrome staining, heart sections were hydrated before 10 min staining in Mayer's hematoxylin (Diapath). Washing in running tap water for 3 min was performed before 5 min incubation in Ponceau 2R (Fisher)/Acid Fuchsin (Fisher) solution (0.35% [m/v] acid fuchsin, 0.7% Ponceau 2R, 1% glacial acetic acid). Heart sections were washed for 1 min in distilled water and then incubated for 7 min in a 1% phosphomolybdic acid solution before 3 min staining in light green (VWR) (1% [m/v] Light greel, 1% glacial acetic acid). Finally, heart sections were washed in 1% glacial acetic acid, then dehydrated by sequential transfer in 80% and 100% ethanol solutions containing 1% glacial acetic acid. Samples were washed one more time with a 100% ethanol solution containing 1% glacial acetic acid before an incubation with xylene and a mounting with Eukitt quick-hardening mounting-medium.

**Immunohistochemistry**

Cryosections (6 µm thickness) were permeabilized for 20 min with 0.5% Triton-X100/PBS before blocking with 1% bovine serum albumin (BSA)/PBS for 30 min. The sections were incubated overnight with primary antibodies against alpha smooth muscle actin (αSMA; 1:300, Sigma) and β-gal (1:6000, Abcam). The sections were then rinsed with PBS, and subjected to nuclear staining with 4′,6-diamidino-2-phenylindole (DAPI) for 45 min (Santa Cruz) at 0.1 μg/mL in 1% BSA/PBS containing Alexa Fluor 488 anti-rabbit and Alexa Fluor 594 anti-chicken secondary antibodies (1:500, Life Technologies). Staining was visualized under the 63× objective lens of Leica TCS SP8 confocal microscope. Images were acquired at a resolution of at least 1024 × 1024.

**RNA extraction and quantitative real-Time PCR (RT-qPCR)**

Total RNA was extracted and purified from cardiac PW1^+^ cells using NucleoSpin RNA XS kit (Macherey-Nagel) following the manufacturer’s protocol. Concentrations and purity were measured by OD260 and OD280 (NanoDrop 1000 Spectrophotometer, Thermo-Fisher Scientific)

For cDNA synthesis, 100 ng of RNA was reversed transcribed using the SuperScript™ IV First-Strand Synthesis System (Thermo-Fisher Scientific). cDNA samples were diluted (1:4) in nuclease-free water before being used in RT-qPCR performed with SYBR Select Master Mix (Life technology) on a Real-Time PCR System (Light Cycler 480, Roche). The master mix (10 µL) contained 5 µL SYBR green, 2.8 µL water, 0.1 µL reverse primers, 0.1 µL forward primers and 2 µL diluted (1:4) cDNA. Appropriate no-RT and non-template controls were included in each 96-well PCR reaction, and dissociation analysis was performed at the end of each run to confirm the specificity of the reaction. The relative *col1a1, aSMA, tgfbr1* and *mmp2* mRNA expression was quantified using the method 2−ΔΔCt. PCR primers details were detailed in supplementary table S2.

**Western blot analysis**

Proteins were extracted from frozen mouse heart tissues with Dounce-Potter homogenizer into ice-cold radioimmunoprecipitation assay (RIPA) buffer (50 mM Tris pH 7.4, 150 mM sodium chloride, 1% IGEPAL CA-630, 50 mM deoxycholate, and 0.1% SDS) supplemented with anti-proteases (Sigma-Aldrich), anti-phosphatase inhibitors (Phosphatase Inhibitor Cocktail 2 and 3, Sigma-Aldrich) and 1 mM sodium orthovanadate. After 1 h incubation at 4°C, the homogenate was centrifuged for 15 min at 15,300 ×*g* and 4°C and the supernatant containing proteins was collected. Protein concentrations for all samples were determined with a Bradford method-based protein assay (Bio-Rad).

After sorting, cardiac PW1^+^ cells from 22 mice and PW1^−^ cells from 16 mice were pooled, centrifuged at 500 ×*g* for 15 min at 4°C, and lysed in urea-thiourea buffer (5 M urea, 2 M thiourea, 50 mM DTT, 0.1% SDS in PBS, pH 7.4). Proteins were extracted as described above. HUVEC cells were lysed in urea-thiourea buffer and proteins were extracted as described above.

Cardiomyocytes (CMs) and non-CMs were isolated from the adult mouse hearts, as previously described with minor modifications ^45^. After cell collection, CMs were lysed in RIPA and urea-thiourea buffers for protein extraction as described above, while the non-CMs were cultured in DMEM supplemented with 10% FBS and 1% P/S. Proteins were extracted using RIPA and urea-thiourea buffer once the cells reached 80% confluency.

Proteins were denatured with NuPAGE LDS sample buffer and NuPAGE Sample Reducing Agent and then loaded on a NuPAGE Novex 4-12% Bis-Tris gel (Life Technologies). After 3 h electrophoresis at 90 V, proteins were transferred onto nitrocellulose membranes using the Trans-Blot Turbo Transfer System (Bio-Rad) and stained with 0.1% Ponceau S (w/v in 5% acetic acid) to assess transfer quality and homogeneous loading. Membranes were blocked for 1 h in Tris-buffered saline with 0.1% Tween-20 (TBS-Tween) containing 5% skim milk with constant shaking and then incubated overnight at 4°C with primary antibodies specific for CD51 (1:2000, Abcam), integrin β1 (1:1000, Abcam), integrin β3 (1:1000, Cell Signaling), integrin β5 (1:500, Cell Signaling), and integrin β8 (1:500, Sigma-Aldrich) diluted in 5% skim milk or BSA/TBS-Tween. After washing, the membranes were incubated for 1 h at room temperature with horseradish peroxidase-labeled secondary antibodies diluted in 5% skim milk/TBS-Tween. Membranes were then washed and incubated for 5 min with SuperSignal West Pico PLUS Chemiluminescent Substrate (Life Technologies) before imaging with the Chemidoc XRS+ camera (Bio-Rad) and analysis using the Image Lab software.

**Supplementary figures**

**Supplementary Figure S1. Co-expression of cell surface markers in cardiac CD45^−^Ter119^−^ and CD45^+^Ter119^−^ cells.** (**A,B**) Expression of CD31 and CD140a (PDGFRα) in cardiac CD45**^−^**Ter119**^−^** (**A**) and CD45^+^Ter119**^−^** cells (**B**).


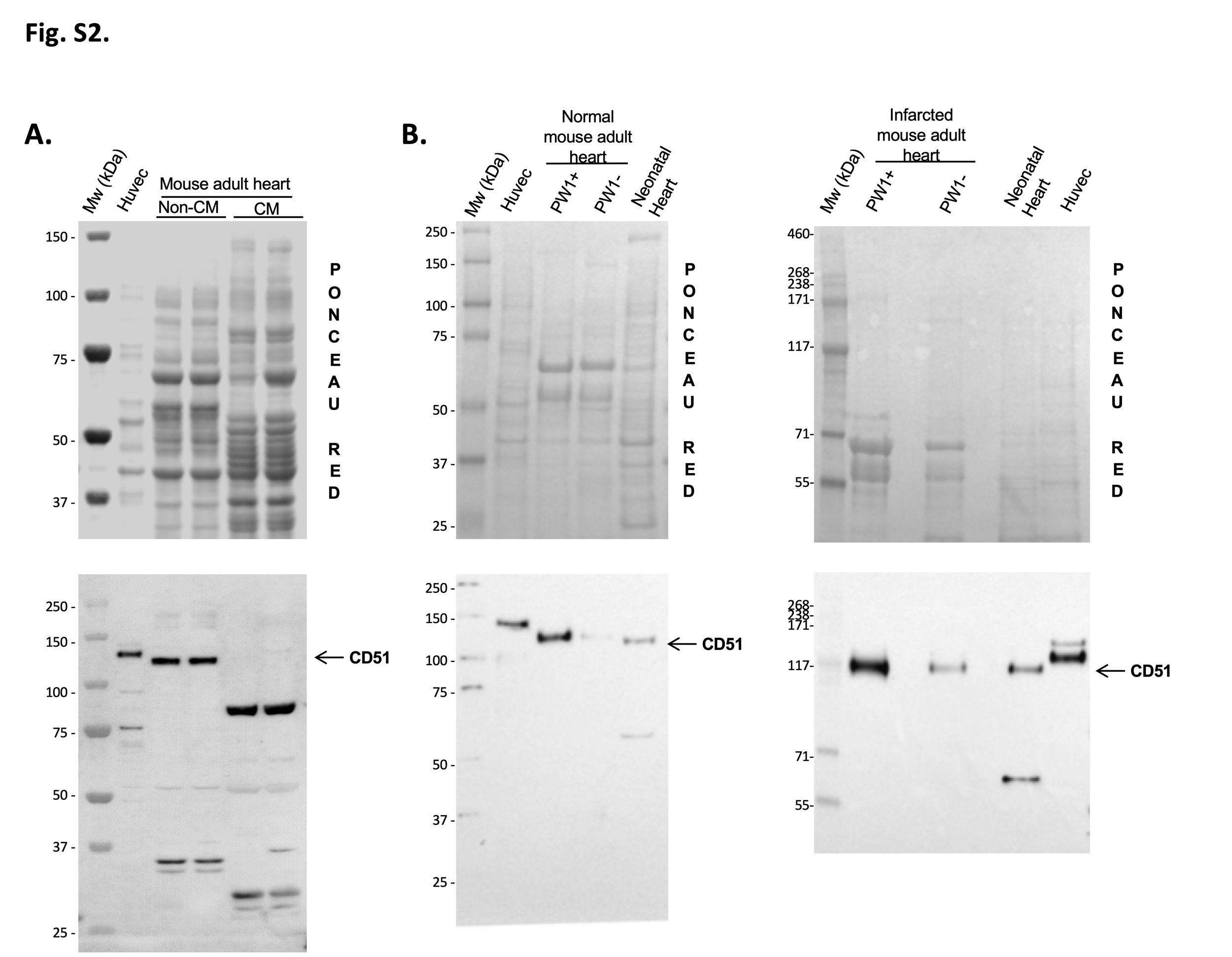


**Supplementary Figure S2. Full length gels and blots corresponding to Figure 3F and G.** (**A**) Cardiomyocytes (CM) and non-CM cells were isolated from wild-type adult mouse hearts and analyzed by western blotting for the expression of CD51. Proteins from HUVEC were used as positive controls. Ponceau S staining showed equal protein loading. (**B**) PW1^+^ and PW1^−^ fractions were FACS-isolated from normal (left panel) and ischemic (7 days post-MI, right panel) hearts of adult PW1^nLacZ^ mice and pooled to analyze the expression of CD51 by western blotting. Proteins from HUVEC and total neonatal hearts were used as controls. kDa, kilodalton; Mw, molecular weight.

**
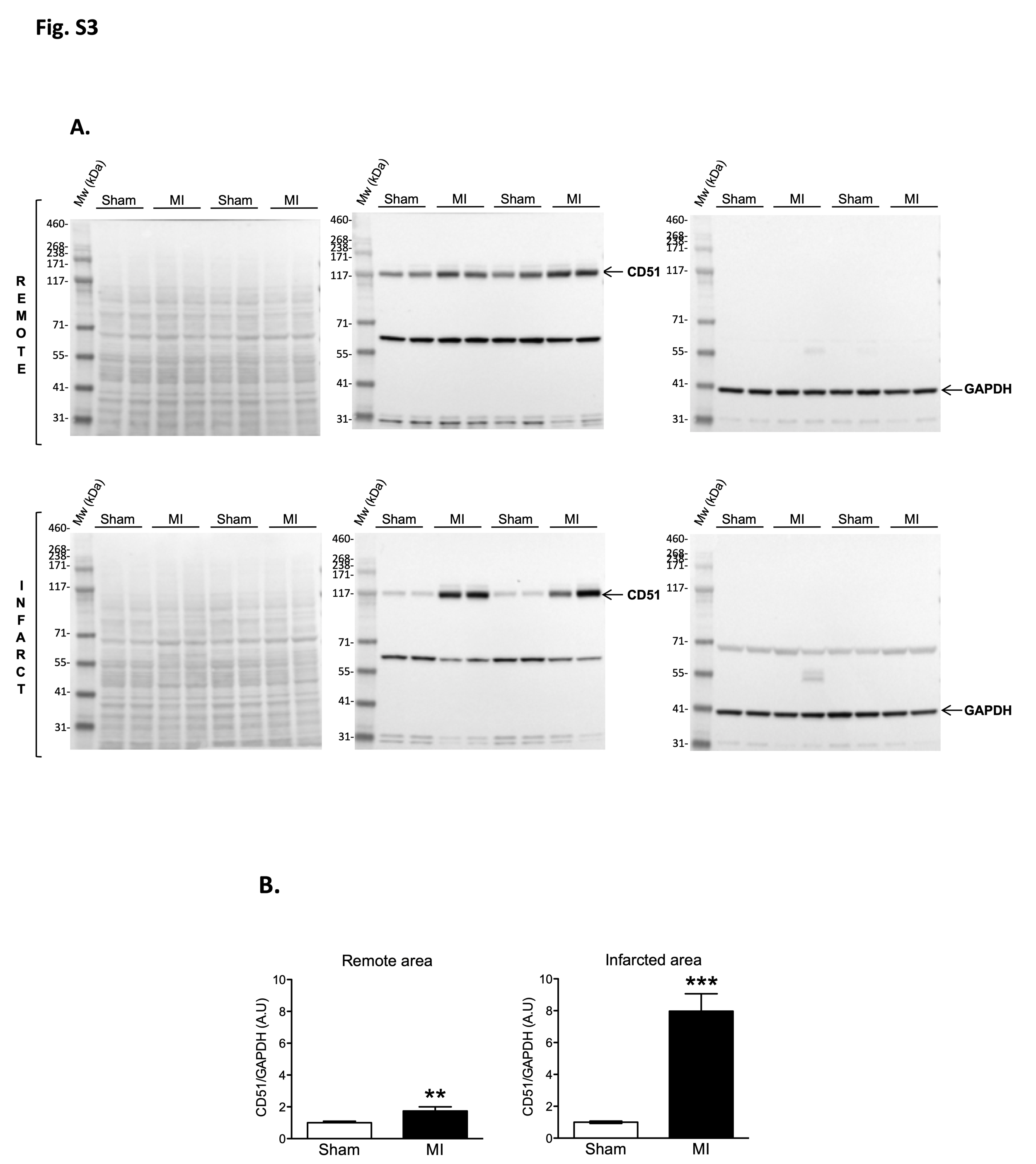
**

**Supplementary Figure S3.** (**A**) **Western blots showing expression of Itgav (CD51) in sham and ischemic hearts** in the remote (upper panel) or infarct (lower panel) zones of ischemic hearts (7 days post-MI) analyzed by western blotting. (**B**) Quantification graphs, Sham n = 7 and MI n = 9, *P < 0.05 and ***P < 0.0001.


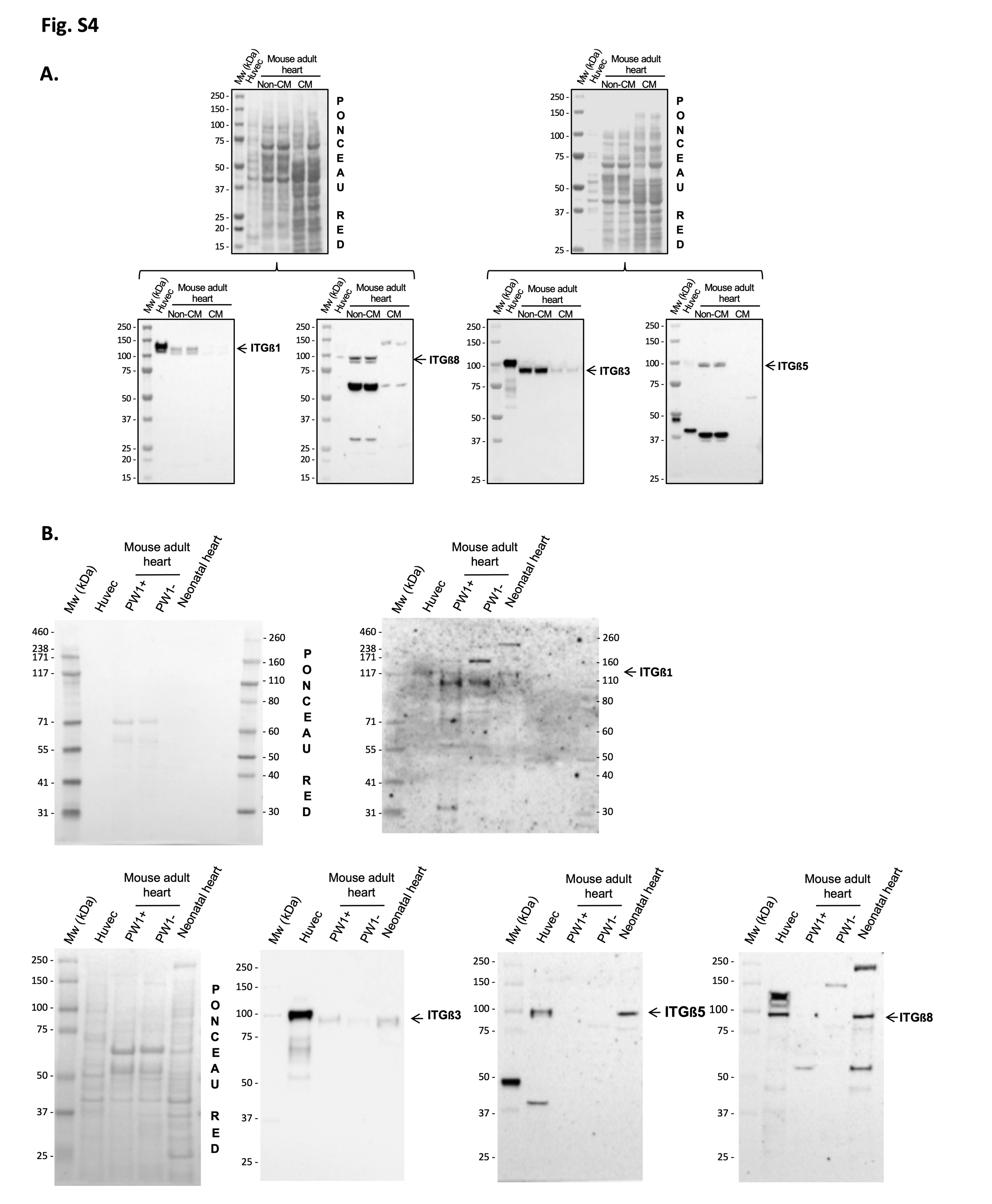
**Supplementary Figure S4. (A)** Expression of ITGβ1, ITGβ3, ITGβ5, and ITGβ8 in freshly isolated cardiomyocytes (CM) and non-CMs. Fractions were isolated from wild-type adult mouse hearts and analyzed by western blotting for the expression of ITGβ1, ITGβ3, ITGβ5, and ITGβ8. Proteins from HUVEC were used as positive controls. (B) Western blots for the presence of ITGβ1, ITGβ3, ITGβ5, and ITGβ8 in PW1^+^ versus PW1^−^ cardiac fractions. Proteins from HUVEC cells and neonatal mouse heart were used as positive controls. Ponceau Red staining showed equal protein loading.

**
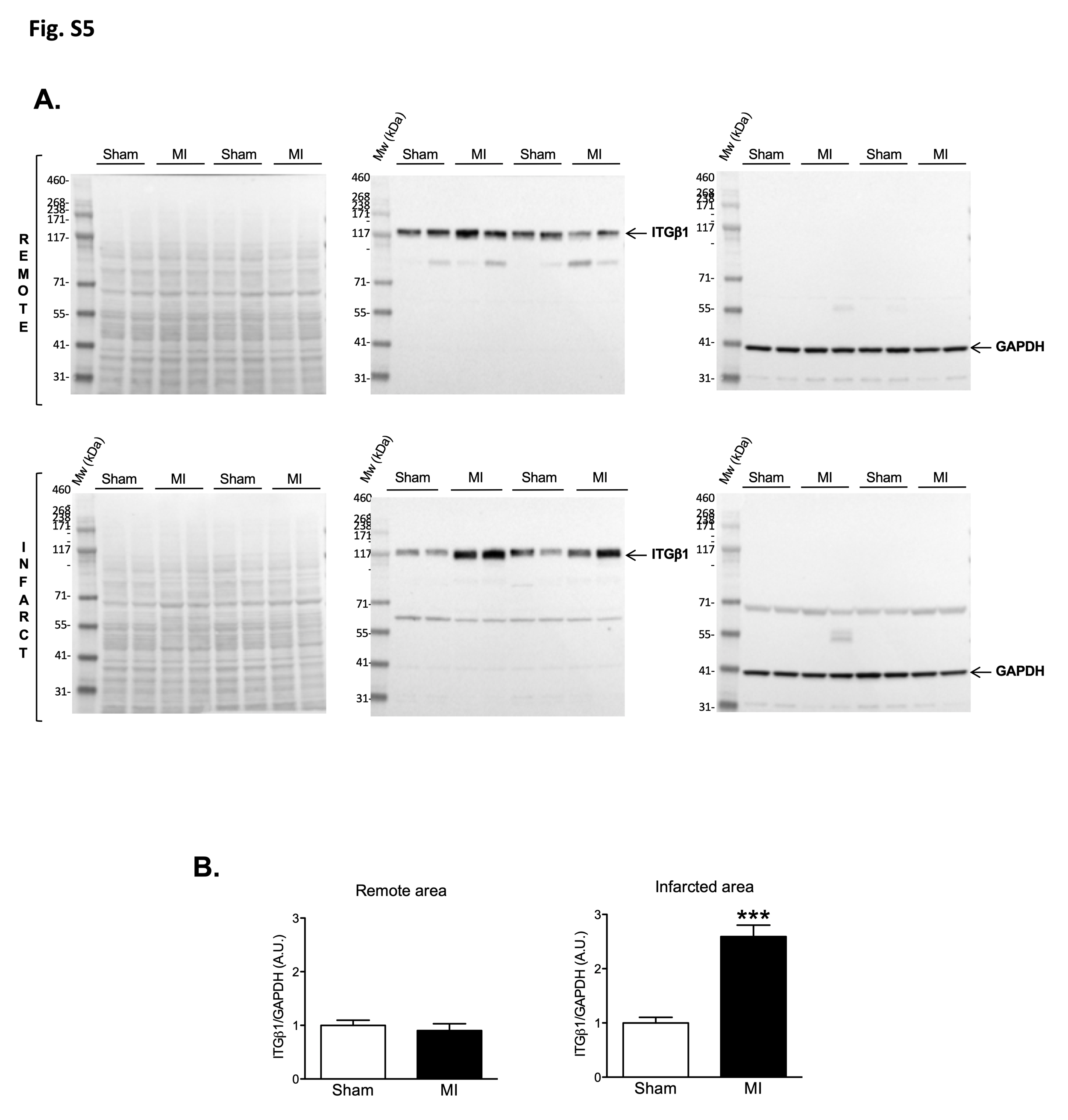
**

**Supplementary Figure S5. Expression of ITGβ1 in sham and ischemic hearts.** **(A)** full length gels and blots showing ITGβ1 expression in sham and the remote (upper left panel) or infarct (lower left panel) zones of ischemic hearts (7 days post-MI), as analyzed by western blotting. **(B)** quantification graphs, Sham n = 7 and MI n = 9, ***P < 0.0001.


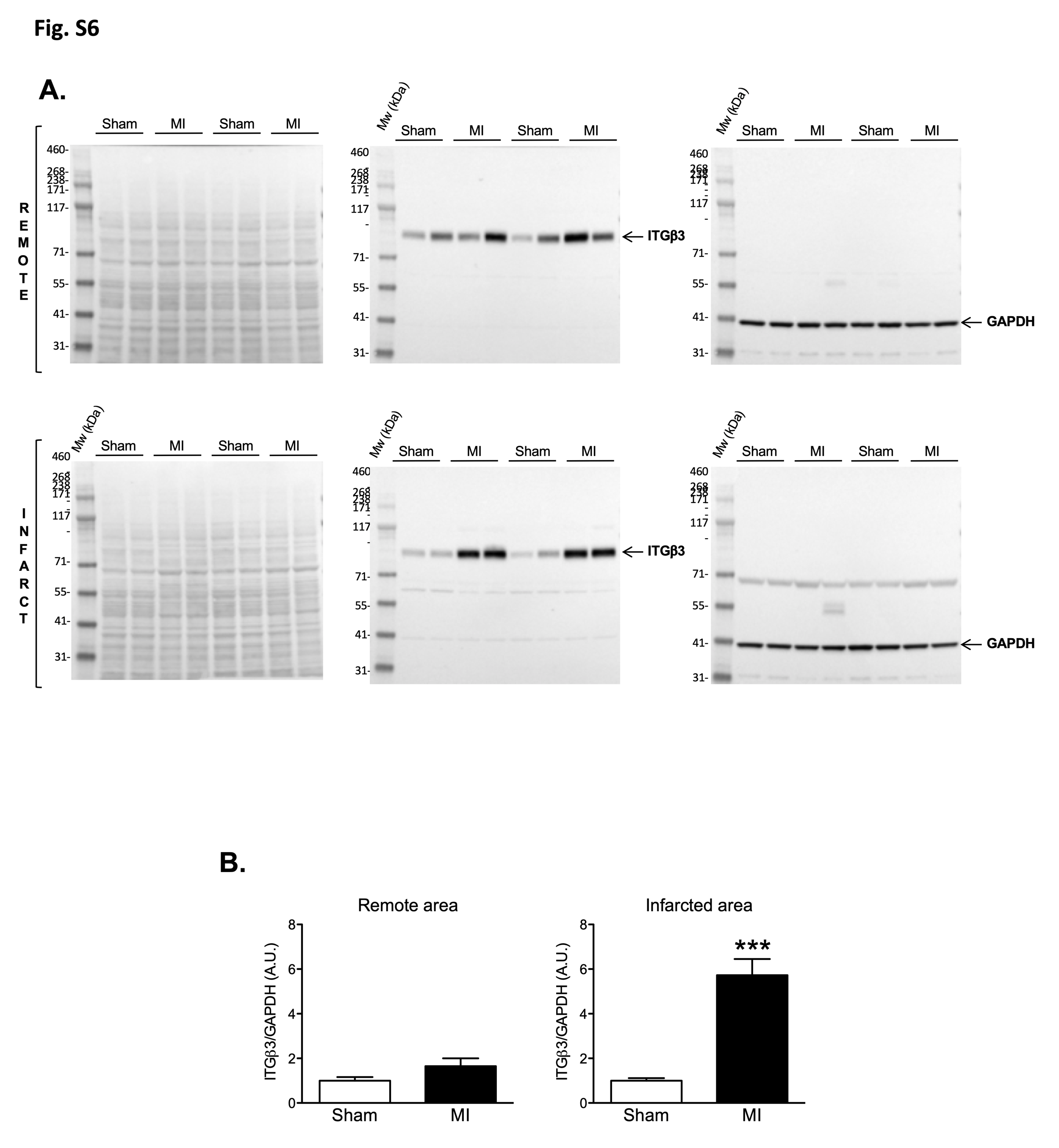


**Supplementary Figure S6. Expression of ITGβ3 in sham and ischemic hearts. (A)** full length gels and blots showing ITGβ3 expression in sham and the remote (upper left panel) or infarct (lower left panel) zones of ischemic hearts (7 days post-MI), as analyzed by western blotting. Right panel: quantification graphs, Sham n = 7 and MI n = 9, ***P < 0.0001.

**Supplementary Figure S7. Cilengitide reduces the expression of profibrotic genes in cardiac PW1^+^ cells.** q-PCR evaluation of *aSMA, mmp2*, *tgfrb1*, and *col1a1* expression in isolated cardiac PW1^+^ cells cultured in presence of cilengitide (0, 300, and 1000 nM). N = 4 in duplicates. *P < 0.05, **P < 0.01, and ***P < 0.001 using Kruskal-Wallis test between all groups; ^#^P < 0.05, ^##^P < 0.01 using Dunn’s comparison with control cells.

**Supplementary Figure S8. No cardiac effect was observed after cilengitide treatment in control groups.** (**A**) Survival curves in cilengitide- versus vehicle-treated mice with a sham procedure. No animal died during the 14 days of survey. (**B**) Cardiac function after 14 days of cilengitide treatment was normal in treated mice. (**C**) Comparison of cardiac fibrosis in vehicle- versus cilengitide-treated mice, showing physiological levels in both groups.

**Supplementary tables**

**Supplementary Table S1.** List of antibodies used for flow cytometry analysis and FACS.

| **Target** | **Fluorophore** | **Clone** | **Supplier** | **reference** |
| --- | --- | --- | --- | --- |
| **CD31** | BUV737 | MEC13.3 | BD Biosciences | 565097 |
| **CD34** | Bv421 | MEC14.7 | Biolegend | 119321 |
| **CD39** | PerCp-Ef710 | 24MDS1 | eBiosciences | 46-0391-82 |
| **CD44** | AF700 | IM7 | eBiosciences | 56-0441-82 |
| **CD45** | PE-Cy7 | 30-F11 | eBiosciences | 25-0451-82 |
| **CD45** | Biotin/PacBlue | 30-F11 | eBiosciences | 13-0451-85 |
| **CD51** | PE | RMV-7 | eBiosciences | 12-0512-83 |
| **CD117** | APC | ACK2 | eBiosciences | 17-1172-83 |
| **CD140a** | APC | APA5 | eBiosciences | 17-1401-81 |
| **CD163** | PE/AF555 | x | Bioss | bs-2527R-A555 |
| **CD166** | PE | eBioALC48 | eBiosciences | 12-1661-82 |
| **CD172a** | AF700 | P84 | biolegend | 144021 |
| **TER119** | BUV395 | Ter-119 | BD Biosciences | 563827 |

**Supplementary Table S2.** List of primers used for q-PCR analysis

| **Gene** | **Forward** | **Reverse** |
| --- | --- | --- |
| ***col1a1*** | AAT-GGC-ACG-GCT-GTG-TGC-GA | AAC-GGG-TCC-CCT-TGG-GCC-TT |
| ***aSMA*** | CAA-TGT-CCC-CGC-CAT-GTA-TG | CAT-CTC-CAG-AGT-CCA-GCA-CA |
| ***tgfbr1*** | AAA-GGT-ACA-TGG-CCC-CTG-AA | ATT-GCA-TAG-ATG-TCA-GCG-CG |
| ***mmp2*** | ACC-CTG-AAA-CCG-TGG-ATG-AT | TGG-ATT-CGA-GAA-AAG-CGC-AG |
